# The effect of synbiotic supplementation on hypothyroidism: A randomized double-blind placebo controlled clinical trial

**DOI:** 10.1371/journal.pone.0277213

**Published:** 2023-02-06

**Authors:** Majid Ramezani, Mahnaz Reisian, Zohreh Sajadi Hezaveh

**Affiliations:** 1 Department of Internal Medicine, School of Medicine, Baqiyatallah University of Medical Sciences, Tehran, Iran; 2 Department of Nutrition, School of Public Health, Iran University of Medical Sciences, Tehran, Iran; Prince Sattam Bin Abdulaziz University, College of Applied Medical Sciences, SAUDI ARABIA

## Abstract

**Objective:**

We hypothesize that synbiotic supplementation could modulate the intestinal microbiota and subsequently, improve the condition of hypothyroid patients.

**Methods:**

Fifty-six adult hypothyroid patients were recruited to this double-blind, placebo-controlled, randomized clinical trial. The intervention was 10 weeks of synbiotic (500 mg of 10^9^ CFU/g probiotics plus fructo-oligosaccharide, n = 28) compared to placebo (lactose, magnesium stearate, talc, and silicon dioxide, n = 28). Randomization and allocation to trial groups were carried out using random number sequences drawn from https://sealedenvelope.com/. Primary outcomes were serum thyroid stimulating hormone (TSH) and free thyroxine (FT4), and secondary outcomes were depression, quality of life, and blood pressure (BP). *P*-values< 0.05 were considered statistically significant.

**Results:**

Analysis on 51 patients who completed the trial showed that TSH and depression (*p*> 0.05) did not change significantly, while serum FT4 significantly increased in both groups (*p* = 0.03 and *p* = 0.02 in symbiotic and placebo respectively). A significant decrease in systolic BP occurred only in the synbiotic group (*p* = 0.05). Significant improvements occurred regarding different domains and areas of quality of life in the crude and adjusted analysis, including perceived mental health (*p* = 0.02), bodily pain (*p* = 0.02), general health perception (*p* = 0.002), and wellbeing (p = 0.002), which were significantly higher in the synbiotic group.

**Conclusions:**

Ten-week supplementation with synbiotic had no favorable effect on depression and TSH, but it improved blood pressure and quality of life in patients with hypothyroidism. More trials are needed to support or reject these findings.

**Trial registration:**

IRCT20210926052583N1, Iranian Registry of Clinical Trials (IRCT), registered October 1^st^, 2021.

## Introduction

Hypothyroidism affects up to 5% of the general population [1], and 7.62 per 1000 individuals in Iran [2, 3]. Since thyroid hormones are essential for normal growth and energy metabolism, thyroid dysfunction can have profound adverse consequences [4]. Despite the increased awareness and the improvements in medical management of this disease, depression, mood disturbance and poor health-related quality of life (QoL) is pretty common among hypothyroid patients [5, 6]. Hypothyroid patients also have reported to have higher blood pressure than their euthyroid counterparts [7]. Lifestyle interventions and non-pharmacologic approaches require several weeks or months to achieve effectiveness. Hence, there is a great need for additional treatment options.

Synbiotics have been advocated as being beneficial to patients with metabolic diseases. Synbiotic refers to a mixture of probiotics and prebiotics that beneficially affect the host by improving the survival and stimulating the growth of advantageous and health promoting microbial species in the gastrointestinal tract [8]. Gut microbiota could be associated with thyroid function [9]. The absorption of essential micronutrients for the thyroid hormone synthesis, including iodine, iron, selenium, zinc and copper is manipulated by the microbiota [10, 11]. The metabolism of iodothyronines (precursor of thyroid hormones), and the intestinal-hepatic cycle of thyroid hormones are also affected by the intestinal flora [12, 13]. Microbiota also has a role in production of inflammatory cytokines [14], which has been found to be related to hypothyroidism [15].

Through the mentioned mechanisms of action, synbiotic plays a major role in the modulation of the intestinal flora, and thus, it could indirectly affect the production of thyroid hormones. Also, according to the literature, synbiotic could improve depression, quality of life [16] and blood pressure [17], all of which are complications of hypothyroidism. As thyroid hormones play a major role in metabolism in various tissues and organs, it is important to address the health and wellbeing of these patients. In the present clinical trial, we tested whether synbiotic supplementation could enhance depression, quality of life, and blood pressure, as well as thyroid hormones in hypothyroid patients, so that we could use synbiotics along with the hormone replacement therapy to improve their condition.

## Methods

### Trial design

This was a 10-week randomized placebo-controlled trial with a parallel design. Participants were recruited from Baqiyatallah hospital through a convenience sampling method between November 2021 and May 2022, and were randomized to the synbiotic (n = 28) or the placebo (n = 28) group. Prior to the start of the trial, the Medical Ethics Committee of Baqiyatallah University of Medical Sciences approved the study (IR.BMSU.BAQ.REC.1400.021; available at: https://ethics.research.ac.ir/ProposalCertificateEn.php?id=206469&Print=true&NoPrintHeader=true&NoPrintFooter=true&NoPrintPageBorder=true&LetterPrint=true). All participants provided written informed consent. Trial registry can be found at the Iranian Registry of Clinical Trials (IRCT) (IRCT20210926052583N1, available at: https://en.irct.ir/user/trial/58952/). The result of this trial is reported according to the Consolidated Standards for Reporting Trials (CONSORT) statement (http://www.consort-statement.org/consort-2010) (see S1 Checklist and S1 Protocol (Persian) and S2 Protocol (English)).

### Participants

The target population was adults with hypothyroidism. Inclusion criteria were: Women or men, 25–65 years old (protocol amendment), with subclinical hypothyroidism (elevated TSH level and a normal or low free T4 level) for at least 6 months (protocol amendment), and BMI less than 35 who were being treated by levothyroxine. Exclusion criteria were: Lactating or pregnancy; History of taking probiotic or synbiotic supplements in the last three months; Smoking or alcohol consumption; Taking appetite suppressants such as antibiotics; Use of antibiotics and any medications that interact with synbiotics; Gastrointestinal diseases such as stomach ulcers, diarrhea or constipation etc. for three months before or during the intervention; Unwillingness to continue cooperation, and non-compliance during the intervention. Participants were also excluded in case of catching infectious diseases such as Covid-19, occurrence of pregnancy, and undergoing surgery during the study period.

### Synbiotic and placebo supplements

Both synbiotic and placebo capsules were provided by Zist-takhmir Pharmacutical company. The synbiotic supplement (Familact, 500 mg) contained Lactobacillus casei, Lactobacillus acidophilus, Lactobacillus rhamnus, Lactobacillus bulgaricus, Bifidobacterium Breve, Bifidobacterium Longum, Streptococcus thermophilus plus fructo-oligosaccharide with microbial population of 10^9^ CFU/g. Placebos contained lactose, magnesium stearate, talc, and silicon dioxide and were made in the same shape, color, odor and taste as the synbiotic supplements.

### Sample size calculation

Sample size was calculated using the G-power software version 3.1.9.2. and data from Talebi et al study [18], considering two-sided first type error α = 0.05, and the second type error equal to 0.20. The primary calculated sample size was 24 individuals. with regard to the expected 15% loss to follow-up, 28 patients were considered in each group (total: 56 patients).

### Study procedures and randomization

Due to the fact that we could not recruit enough participants through flyers and advertising, the researcher attended the hospital where the sampling took place, and interviewed the eligible participants, who were referred to the researcher by the practitioners according to the inclusion criteria. After explaining the aims and circumstances of the trial, eligible patients who opted for participation were asked to sign a written informed consent. Baseline data including demographics, anthropometric indices, blood pressure, and questionnaires including Short Form Health Survey (SF-36) and Beck Depression Inventory II (BDI-II) were collected. They were then asked to refer to the hospital’s laboratory for the blood sample collection (the same day if they were fast for 8–12 hours, and if not, the day after, before taking the supplements) (protocol amendment). This process was repeated after 10 weeks of supplementation.

Details on randomization is reported in the published protocol of this trial [19]. Block randomization method with a block size of 4 was used. Random number sequences were drawn from https://sealedenvelope.com/. For allocation concealment, unique codes were written on supplement boxes. Participants, researchers, practitioners, and outcome assessor were blinded to the treatment allocation.

All participants received a box of capsules (synbiotic or placebo) at first visit based on randomization. The researcher also educated the participants on the time and amount of supplement intake (1 capsule daily after lunch or dinner), as well as possible complications. Dietary and physical activity recommendations were also provided (details available in the protocol [19]). Participants were contacted every week via telephone or social media and asked about adherence to the study protocol, changes in medication, or any possible side effects.

### Outcomes

Serum levels of TSH and fT4 were assessed as the primary outcomes, using Eliza kits (Pishtazteb Tehran, Iran). The secondary outcomes were systolic (SBP) and diastolic blood pressure (DBP) which were assessed by a digital barometer (Omron, Tokyo, Japan), quality of life (QoL) which was measured using SF-36 questionnaire (including 8 domains and 3 areas) [20], and depression which was assessed using BDI-II (including 2 subscales) [21, 22]. The information on anthropometric indices, physical activity (International Physical Activity Questionnaire (IPAQ-SF)), and dietary intake (24-hour dietary recall for 2 weekdays and 1 weekend) were also collected to control the potential confounders and covariates. Waist circumference was not measured, as the patients insisted on social distancing (protocol amendment). Details on data collection is present in the protocol of this trial [19].

### Statistical analysis

Normality of data was checked by the measures of central tendency, measures of dispersion and P-P and Q-Q plots. All variables are reported as mean ± SD. Baseline characteristics of quantitative variables were compared between the two groups by independent sample t-test. The difference between the two groups regarding qualitative variables were assessed by Chi-square test or its alternative, Fisher’s exact test. Analysis of Covariance (ANCOVA) was performed to eliminate the effect of confounders. Changes from the baseline to post-intervention within each group were assessed through a paired t-test. A *p*-value≤ 0.05 will be considered statistically significant. Data were analyzed using SPSS software (IBM Corp. Released 2016. IBM SPSS Statistics for Windows, Version 24.0. Armonk, NY: IBM Corp). GraphPad Prism software was used to draw graphs (GraphPad Prism version 8.0.0 for Windows, GraphPad Software, San Diego, California USA, www.graphpad.com).

## Results

**Fig 1:**
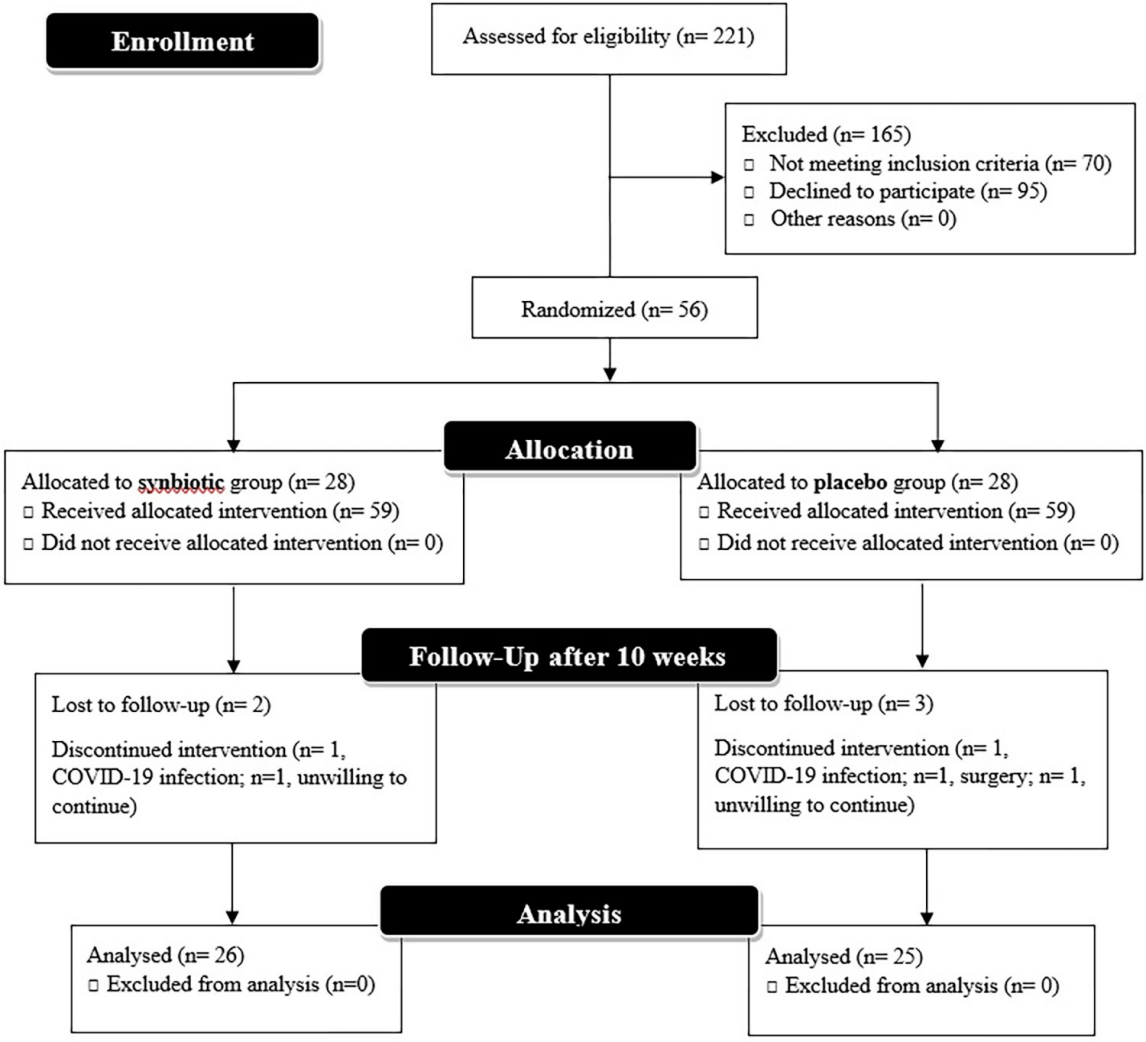
CONSORT flow diagram of participant recruitment and intervention.

**Fig 1** depicts the process of participant recruitment and allocation. Sampling was performed between November 2021 and May 2022. Of 56 eligible and interested hypothyroid patients, 2 patients from the synbiotic group (1 COVID-19 infection and 1 unwilling to continue) and 3 from the placebo group (1 COVID-19 infection, 1 surgery, and 1 unwilling to continue) withdrew the study, and the study was completed with 51 patients (26 in the synbiotic and 25 in the placebo group). Positive feedback was received from the patients and they reported no adverse effects. The adherence to supplement intake was calculated to be 91.37%.

Evaluation of the baseline characteristics of the patients is represented in **Table 1**. There were a few missing data regarding the baseline characteristics, which is reported in detail in **S1 Table**. The mean age of participants was 47.48 ± 9.14 years (48.89 ± 9.45 in the synbiotic and 46.07 ± 8.76 in the placebo group). Most participants in both groups were female (~80%). Participants of both groups were mostly married, overweight and had autoimmune hypothyroidism. As requested, patients did not change their dietary intake and physical activity throughout the study period (**Table 2**). Baseline intake of carbohydrate was significantly different between the two groups (*p* = 0.02), however, this statistical significancy was ignored according to the CONSORT guideline (only clinical significancy should be taken into account regarding the baseline characteristics). On the other hand, the difference between the two groups in terms of age, dose of levothyroxine, duration of hypothyroidism, and physical activity were considered clinically significant, and were adjusted in the ANCOVA analysis.

**Table 1:** Comparison of baseline characteristics of the participants.

Variable		Synbiotic (n = 28)	Placebo (n = 28)	P-value
**Age (year)**		48.89 ± 9.45	46.07 ± 8.76	0.25
**Weight (Kg)**		75.15 ± 15.61	77.14 ± 17.16	0.65
**Height (cm)**		1.63 ± 0.07	1.63 ± 0.08	0.98
**BMI (kg/cm** ^**2**^ **)**		28.05 ± 4.98	28.79 ± 5.30	0.59
**Levothyroxine dose (μg)**		77.51 ± 25.50	88.28 ± 21.12	0.10
**Duration of Hypothyroidism (year)**		9.50 ± 6.97	12.59 ± 10.69	0.21
**Sex** ^a^	**Female**	24 (85.7%)	23 (82.1%)	1.00
	**Male**	4 (14.3%)	5 (17.9%)	
**Type of Hypothyroidism** ^a^	**Autoimmune**	19 (79.2%)	19 (90.5%)	0.27
	**Non-autoimmune**	5 (20.8%)	2 (9.5%)	
**Education**	**Under diploma**	8 (28.6%)	5 (17.9%)	0.36
	**Diploma**	12 (42.9%)	10 (35.7%)	
	**University**	8 (28.6%)	13 (46.4%)	
**Marita Status** ^a^	**Married**	23 (82.1%)	21 (75.0%)	0.88
	**Single**	4 (14.3%)	4 (14.3%)	
	**Divorced**	0 (0.0%)	1 (3.6%)	
	**Widowed**	1 (3.6%)	2 (7.1%)	
**Vitamin-mineral supplement intake**	**Yes**	15 (53.6%)	14 (50.0%)	0.79
	**No**	13 (46.4%)	14 (50.0%)	

Independent sample t-test or chi-square test

^a^ Two-tailed Fisher’s exact test

Mean ± SD or n (%)

**Table 2:** Physical activity and dietary intake of the participants before and after the intervention.

Variable		Synbiotic (n = 28)	Placebo (n = 28)	P-value
**Physical activity (MET-minute/week)**	**Before**	583.04 ± 829.08	389.95 ± 552.09	0.31
	**After**	430.15 ± 583.13	294.18 ± 509.63	0.38
**Calorie (Kcal)**	**Before**	1373.01 ± 265.95	1428.53 ± 249.87	0.43
	**After**	1403.09 ± 291.49	1368.28 ± 188.29	0.61
**Protein (gr)**	**Before**	57.65 ± 23.58	63.61 ± 18.45	0.30
	**After**	58.22 ± 15.54	58.53 ± 17.57	0.95
**Carbohydrate (gr)**	**Before**	212.70 ± 62.88	265.69 ± 96.91	0.02
	**After**	226.46 ± 70.86	243.68 ± 72.48	0.39
**Fat (gr)**	**Before**	59.82 ± 21.68	74.10 ± 48.12	0.16
	**After**	61.39 ± 29.42	61.39 ± 29.42	0.99
**Fiber (gr)**	**Before**	12.17 ± 4.85	13.75 ± 5.54	0.26
	**After**	11.52 ± 5.03	12.77 ± 5.17	0.38

Independent sample t-test; Mean ± SD.

The result of the two-way ANCOVA analysis is provided in **Table 3**. Neither within group, nor between group changes were observed for serum TSH level following supplementation (*p*> 0.05). But the amount of FT4 increased significantly in both groups (mean change: 2.43 ± 5.03, P = 0.03; and 2.87 ± 5.80, *p* = 0.02 respectively for the synbiotic and placebo group). However, ANCOVA analysis showed no difference between the two groups before and after the 10 weeks (*p* = 0.75).

In terms of depression, although a reduction was observed in the synbiotic group (mean change: -1.15 ± 5.04) and an increase was observed in the placebo group (mean change: 0.04 ± 6.25), the observed changes were not significant (*p*> 0.05). This applies to the somatic and affective subscales of depression as well (*p* = 0.15 and *p* = 0.27 respectively).

**Table 3:** Primary and secondary outcomes before and after the intervention in synbiotic and placebo groups.

	Variable		Before	After	Change	P-value^1^
**Thyroid Hormones**	**Free T4 (μg/dl)**	**Synbiotic (n = 26)**	9.47 ± 2.97	12.05 ± 3.96	2.43 ± 5.03	0.03
		**Placebo (n = 25)**	10.59 ± 3.62	13.63 ± 6.75	2.87 ± 5.80	0.02
		**P-value** ^**2**^	0.22	0.33	0.88	
		**P-value** ^**3**^	0.75			
	**TSH (μIU/ml)**	**Synbiotic (n = 26)**	1.73 ± 1.29	1.49 ± 1.14	-0.05 ± 0.74	0.74
		**Placebo (n = 25)**	2.27 ± 1.86	2.17 ± 1.74	-0.05 ±1.95	0.87
		**P-value** ^**2**^	0.23	0.12	0.99	
		**P-value** ^**3**^	0.39			
**Depression**	**Total Depression**	**Synbiotic (n = 26)**	16.11 ± 8.45	15.58 ± 9.52	-1.15 ± 5.04	0.25
		**Placebo (n = 25)**	15.11 ± 9.50	15.52 ± 8.29	0.04 ± 6.25	0.97
		**P-value** ^**2**^	0.68	0.98	0.46	
		**P-value** ^**3**^	0.11			
	**Somatic**	**Synbiotic (n = 26)**	11.77 ± 6.51	10.42 ± 6.50	-1.35 ± 4.33	0.13
		**Placebo (n = 25)**	11.64 ± 6.82	11.48 ± 5.85	-0.16 ± 4.23	0.85
		**P-value** ^**2**^	0.95	0.54	0.33	
		**P-value** ^**3**^	0.15			
	**Affective**	**Synbiotic (n = 26)**	4.96 ± 3.28	3.84 ± 3.89	0.19 ± 1.90	0.61
		**Placebo (n = 25)**	5.15 ± 3.78	4.04 ± 3.35	0.20 ± 2.91	0.73
		**P-value** ^**2**^	0.35	0.27	0.99	
		**P-value** ^**3**^	0.27			
**Quality of life**	**Physical Function**	**Synbiotic (n = 26)**	64.46 ± 23.54	74.81 ± 21.52	9.61 ± 22.13	0.36
		**Placebo (n = 25)**	74.29 ± 23.08	69.62 ± 25.86	-2.60 ± 20.92	0.54
		**P-value** ^**2**^	0.12	0.44	0.051	
		**P-value** ^**3**^	0.18			
	**Role Limiting Physical Function**	**Synbiotic (n = 26)**	55.36 ± 43.76	69.60 ± 25.86	17.31 ± 41.09	0.04
		**Placebo (n = 25)**	72.32 ± 34.92	65.00 ± 37.50	-4.00 ± 34.37	0.57
		**P-value** ^**2**^	0.11	0.68	0.053	
		**P-value** ^**3**^	0.14			
	**Role Limiting Emotional Function**	**Synbiotic (n = 26)**	48.51 ± 42.67	53.52 ± 41.91	5.13 ± 60.14	0.67
		**Placebo (n = 25)**	67.26 ± 38.62	60.67 ± 39.93	-2.67 ± 41.85	0.75
		**P-value** ^**2**^	0.09	0.54	0.60	
		**P-value** ^**3**^	0.60			
	**Vitality**	**Synbiotic (n = 26)**	55.36 ± 19.00	58.65 ± 17.51	3.60 ± 13.13	0.17
		**Placebo (n = 25)**	59.15 ± 20.37	53.25 ± 23.18	-4.25 ± 12.06	0.09
		**P-value** ^**2**^	0.47	0.35	0.03	
		**P-value** ^**3**^	0.07			
	**Perceived Mental Health**	**Synbiotic (n = 26)**	57.36 ± 22.16	60.58 ± 19.15	4.81 ± 13.75	0.09
		**Placebo (n = 25)**	59.15 ± 20.37	55.00 ± 21.11	-2.80 ± 12.08	0.26
		**P-value** ^**2**^	0.71	0.33	0.04	
		**P-value** ^**3**^	0.02			
	**Social Function**	**Synbiotic (n = 26)**	56.70 ± 23.93	65.86 ± 18.22	7.69 ± 23.47	0.11
		**Placebo (n = 25)**	74.73 ± 22.75	74.30 ± 23.12	0.60 ± 20.50	0.88
		**P-value** ^**2**^	0.01	0.15				0.26	
		**P-value** ^**3**^	0.15			
	**Pain**	**Synbiotic (n = 26)**	57.41 ± 24.22	63.27 ± 23.77	8.27 ± 21.07	0.052
		**Placebo (n = 25)**	71.43 ± 28.36	65.90 ± 26.91	-3.30 ± 18.39	0.38
		**P-value** ^**2**^	0.005	0.71				0.04	
		**P-value** ^**3**^	0.02			
	**General Health Perception**	**Synbiotic (n = 26)**	55.18 ± 19.36	62.69 ± 15.89	7.88 ± 14.43	0.01
		**Placebo (n = 25)**	64.29 ± 19.71	58.60 ± 18.23	-4.20 ± 12.64	0.11
		**P-value** ^**2**^	0.09	0.40	0.003	
		**P-value** ^**3**^	0.002			
	**Health Change**	**Synbiotic (n = 26)**	50.89 ± 24.04	59.62 ± 23.53	9.61 ± 30.88	0.12
		**Placebo (n = 25)**	47.32 ± 24.85	46.00 ± 23.58	1.00 ± 13.46	0.71
		**P-value** ^**2**^	0.59	0.04	0.21	
		**P-value** ^**3**^	0.59					
**Blood pressure**	**SBP (mmHg)**	**Synbiotic (n = 26)**	120.89 ± 12.77	118.46 ± 9.14				-2.50 ± 6.36	0.05
		**Placebo (n = 25)**	124.07 ± 11.52	123.65 ± 9.33				-0.60 ± 4.41	0.50
		**P-value** ^**2**^	0.34	0.05				0.22	
		**P-value** ^**3**^	0.44			
	**DBP (mmHg)**	**Synbiotic (n = 26)**	78.21 ± 8.95	75.96 ± 6.33				-2.11 ± 5.51	0.06
		**Placebo (n = 25)**	80.38 ± 8.36	81.35 ± 10.15				1.20 ± 8.45	0.48
		**P-value** ^**2**^	0.36	0.03				0.10	
		**P-value** ^**3**^	0.28			

1: within group comparison, paired t-test

2: between group comparison, independent t-test

3: two-way ANCOVA analysis with adjustment for duration of hypothyroidism, age, levothyroxine dose, and physical activity

**Fig 2:**
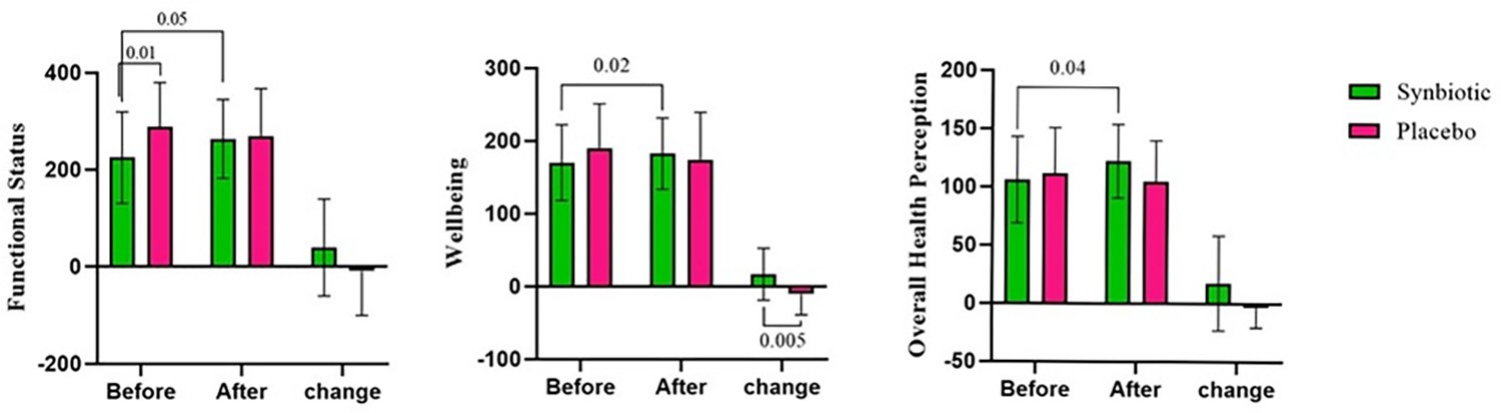
Comparison of the areas of quality of life before and after the intervention in synbiotic and placebo groups. Data represented for 26 participants in the synbiotic and 25 participants in the placebo group. p-values ≤ 0.05 (significance level) are shown.

Significant results were observed for most of the domains of the QoL. The change in physical functioning (*p* = 0.051), role limitations due to physical problems (*p* = 0.053), bodily pain (*p* = 0.04), general health perceptions (*p* = 0.003), vitality (*p* = 0.03), and perceived mental health (*p* = 0.04) were significantly different between the two groups. This significancy remained unchanged after adjustment for confounding variables regarding perceived mental health (*p* = 0.02), bodily pain (*p* = 0.02), and general health perceptions (*p* = 0.002). After 10 weeks of supplementation, pain and general health perception were remarkably improved in the synbiotic group (*p* = 0.052, *p* = 0.01 respectively), while such change did not occur in the placebo group. The three areas of the SF-36 questionnaire were also compared between the two groups before and after the supplementation (**Fig 2**). Remarkable improvements occurred in terms of functional status, wellbeing and overall health evaluation (*p* = 0.051, *p* = 0.02, and *p* = 0.04 respectively) only in the synbiotic group. ANCOVA analysis revealed a remarkable enhancement in the wellbeing area in both adjusted (*p* = 0.002) and non-adjusted (*p* = 0.005) models.

In terms of blood pressure, both SBP and DBP were significantly different between the synbiotic and the placebo group after the intervention (*p* = 0.05 and *p* = 0.03 respectively). A significant reduction was observed in the synbiotic group (*p* = 0.05), while such reduction was not significant in the placebo group (*p* = 0.06). Merging the effect of time and group together, the ANCOVA analysis showed no significant difference between the two groups in both the adjusted and non-adjusted models.

We also performed the Intention-to-treat (ITT) analysis due to the loss of sample size (**S2 Table**). The results were mostly similar to that of per protocol analysis, with a slight difference regarding QoL. In the ITT analysis, social functioning was significantly different between the two groups in the adjusted ANCOVA model (*p* = 0.03), while the change in the physical functioning score turned non-significant (*p* = 0.06).

## Discussion

In this double-blind randomized controlled trial, the effect of synbiotic on complications of hypothyroidism was investigated. According to the findings of the present study, 10 weeks supplementation with 500 mg of 10^9^ CFU/g probiotics plus fructo-oligosaccharide in comparison to placebo could not affect serum TSH level and depression, while it significantly improved blood pressure and various domains and areas of QoL. The fact that this effect remained significant even after adjustment for confounders shows that the potential confounders had little impact on the estimates of treatment effects. This supplementation was well tolerated and no complication was reported by the participants.

Although significant improvement in serum FT4 level was observed in the within group analysis, the result of between group comparison and ANOVA analysis showed no significant changes. TSH level also remained unchanged after the intervention. This finding is in accordance with that of Talebi et al. [18]. They reported no significant changes in serum TSH level following 8 weeks of synbiotic supplementation. Similarly, Spaggiari et al. demonstrated that treatment with a mixture of probiotics (VSL#3) did not significantly alter serum thyroid hormones in hypothyroid patients [23]. On the contrary, Narimani-Rad et al. [24]reported lower TSH and greater T4 levels in athletes in the probiotic group after 30 days of supplementation. Microbiota is involved in iodothyronines metabolism [25], converting T4 to T3 [26], and absorption of oral thyroxine [12], and since synbiotic modulates the gut microbiota, a significant improvement was expected regarding thyroid hormones in the present study. However, the non-significant result could be attributable to two reasons. Firstly, the majority of included patients in this trial had autoimmune hypothyroidism. The impact of gut microbiota on innate immunity mostly occurs during the infancy [27, 28], hence, maybe the modification of intestinal microbial load by synbiotics in adulthood is not effective enough to alter the source and characteristics of autoimmune hypothyroidism. Secondly, serum level of thyroid hormones and TSH is mostly affected by the levothyroxine treatment [1]. In the present study, the practitioners made no changes to the dose of levothyroxine medication, and hence, no significant changes occurred in FT4 and TSH.

Depression was not significantly affected by the intervention in the present study. Observational studies have reported contradictory results regarding the existence of depression in hypothyroid patients [29, 30]. Synbiotic supplementation was also more effective than probiotic in improving depression in hemodialysis patients [31]. However, this trial was 12 weeks long, and depression was assessed using the Hospital Anxiety and Depression Scale (HADS).

Participants of this trial had an average BDI score of ~15, which is categorized as mild depression (score = 14–19). It is evident that synbiotic supplementation affects depression in major depressive disorder and severe depression [32]. On the other hand, in trials with significant reduction in depression score following synbiotic supplementation, synbiotic was used in combination with either antidepressants such as fluoxetine [33] or plant extracts and nutrients [34]. Therefore, maybe synbiotic affects depression in hypothyroid patients, if they have moderate or severe form of depression, and if it is co-supplemented with other depression reducing agents.

QoL was significantly improved in hypothyroid patients after synbiotic consumption in almost all domains and areas, except for role limitations due to emotional problems. Unfortunately, our results are incomparable to that of other trials, as previous clinical trials in this regard have been performed among cancer patients [35] or patients with gastrointestinal diseases [36], whose quality of life is mostly affected by their disease activity and severity. Another important point to consider, is that this study was performed during the COVID-19 pandemic. Although the number of new cases and the death rates were rather low in Iran during the sampling period [37], it still was a huge public concern.

Health-related QoL is a multi-dimensional concept that implies the subjective assessment of different aspects of life and shows a person’s perception of the impact of ill health on daily life [38]. Altering levothyroxine doses in hypothyroid patients to keep TSH levels in and near the reference range does not improve quality of life [39], and it is lower in treated hypothyroid patients than in general population [40]. In other words, satisfactory laboratory measurements should not be judged exclusively, as QoL determines patients’ wellbeing, impacting their overall life and their satisfaction with the therapy [41]. Improvement in QoL is a crucial outcome of a successful therapy. Given the fact that hypothyroidism therapy is a lifelong intervention, using synbiotic supplement seems to satisfy patients’ well-being and convenience.

In the present trial, administration of synbiotic for 10 weeks significantly reduced SBP and DBP. Probiotics have been reported to exert Angiotensin-converting enzyme (ACE)-inhibitory activity and play a role in γ-Aminobutyric acid (GABA) production, both of which lead to a reduction in blood pressure [42]. Our finding is in line with that of Asemi et al. who found significant reduction in blood pressure in patients with type 2 diabetes following probiotic supplementation [43]. However, 8 weeks of synbiotic consumption showed no favorable effect on blood pressure in hypothyroid patients [18]. The researchers blamed the non-significant result on the insufficient dose of synbiotic supplement. However, as we observed significant reduction, maybe the duration of intervention is an important factor. Also, the baseline values of both SBP and DBP were around 1 mmHg higher in participants of the present study, so maybe synbiotic is more effective in reducing higher levels of blood pressure.

### Strengths

This is the first clinical trial done on synbiotic for quality of life and depression in the hypothyroid patients. Number of patients lost to follow up and patients with poor compliance were minimal, confirming patient reports of high acceptability and increasing the statistical power of the findings. Also, the ITT analysis allowed all participants who were randomized to be included in the statistical analysis. Random assignment of treatment groups allowed for controlling for regression to the mean as an explanation for the apparent treatment effects. Enrolling patients over a short period of time (7 months) minimized the effects of seasonal changes in depression. It also limited the effect of the ups and downs in the prevalence of COVID-19, on the results. The use of placebo and blinding lead to a clearer understanding of the mechanism of action of the intervention. This research was also designed according to the SPIRIT guideline and reported according to the CONSORT checklist.

### Limitations

One limitation is the generalizability of the results. Although Baqiyatallah is a referral hospital and the sample was socially, culturally and geographically diverse, we had plenty of inclusion and exclusion criteria. Several protocol amendments had to be made, because of the low response rate to the advertisement and fliers, and difficulty in reaching the final sample size. Another limitation was adjustment for 4 confounders in the ANCOVA analysis. Since the sample size was small (less than 30 patients in each group), adjusting 4 confounders widened the 95% confidence interval (CI) values, and lead to a less accurate result. We also failed to perform subgroup analyses (i.e. based on type of hypothyroidism or age groups) to find out the reason for non-significant results regarding thyroid hormones and depression, because of the small sample size. Measurement of the richness and diversity of gut microbiota was outside the scope of this study; hence, it is inconclusive through which mechanisms synbiotic supplementation has induced the observed changes. Budget limitation also prevented us from assessing more important outcomes including serum T3 level.

### Future directions of the study

It could be suggested that future studies with bigger sample sizes, longer durations, and different types and doses of probiotics and prebiotics further assess this effect in hypothyroid patients. The effect of synbiotics and probiotics with various content of pro- and prebiotics could be assessed on other important aspects of hypothyroidism, such as fatigue, cardiovascular status, etc. Assessment of the microbiota and its metabolites could also clarify the mechanism through which synbiotics affect these patients’ health.

### Conclusion

This clinical trial led to the conclusion that 10 weeks supplementation with synbiotics might be effective in enhancing the QoL and blood pressure in hypothyroid patients, although it may not improve depression and serum TSH level. More clinical trials are required to assess the effectiveness of this supplementation along with the routine treatment for hypothyroid patients.

## Supporting information

S1 Checklist CONSORT checklist. (DOC)

S1 Protocol Persian protocol. (PDF)

S2 Protocol English protocol. (DOCX)

S1 Table Number of missing data at baseline. (DOCX)

S2 Table ITT analysis of the outcomes. 1: within group comparison, paired t-test, 2: between group comparison, independent t-test, 3: two-way ANCOVA analysis with adjustment for duration of hypothyroidism, age, levothyroxine dose, and physical activity.
(DOCX)

S1 Visual abstract (DOCX)
