# Supplementary material for: The effect of synbiotic supplementation on hypothyroidism: A randomized double-blind placebo controlled clinical trial
Source: PLoS One. 2023 Feb 6;18(2):e0277213. doi: 10.1371/journal.pone.0277213 (PMC9901790; doi:10.1371/journal.pone.0277213)
Supplement: S1 Protocol — (PDF) [file pone.0277213.s002.pdf]

بسمه تعالی

با سلام واحترام خواهشمند است پس از تکمیل موارد زیر شناسنامه را به مدیریت پژوهش تحویل نمایید :

1- یک نسخه از مقالات استفاده شده در شناسنامه (فقط صفحه Abstract) تهیه و ضمیمه شناسنامه گردد.

2- یک نسخه از پرسشنامه جمع آوری اطلاعات می بایست ضمیمه شناسنامه شود.(در صورت استفاده از پرسشنامه در طرح تحقیقاتی)

3- کلیه صفحات شناسنامه می بایست توسط سرپرست طرح امضاء شود.

4- جداول گانت ، مجموع بودجه های طرح ، فرم عملیات اجرایی ، جدول همکاران می بایست تکمیل گردد.

5- فرم شماره 11 (فرم مشخصات سرپرست پژوهشگران ،همکاران....)توسط تمامی همکاران طرح امضا شود.

6- یک حلقه CD محتوی شناسنامه اصلاح شده در قالب word وهمچنین فایل پرسشنامه وفایل مقالاتی که به عنوان رفرنس استفاده شده اند بایستی ضمیمه شود.(این کار پس از تصحیح اشکالات شناسنامه بایستی صورت گیرد.)

با عرض پوزش در صورت رعایت نشدن موارد فوق از تحویل گرفتن پروپوزال معذوریم.

باتشکر

مدیر پژوهش دانشکده پزشکی

دکترزهره نادری

« بسمه تعالي »

دانشگاه علوم پزشکی بقیه الله (عج)

شنـــاسنـــامـــه طــرح تحقیقـــاتـــی

عنوان : تاثیر مکمل یاری با سینبیوتیک بر هرمون های تیروئیدی، فشار خون، افسردگی و کیفیت زندگی در بیماران دچار کم کاری تیروئید

سرپرست : دکتر مجید رمضانی

مرکز ارائه‌دهنده : دانشکده پزشکی

شناسه طرح	

گردشگار تصویب طرحهای تحقیقاتی

ردیف	مرحله	تاریخ	تحویل گیرنده	امضا و ملاحظات
1	تحویل عنوان طرح به دانشکده یا مرکز تحقیقاتی			
2	تصویب عنوان طرح در دانشکده یا مرکز تحقیقاتی			
3	تحویل شناسنامه طرح به دانشکده یا مرکز تحقیقاتی			
4	تصویب طرح در شورای پژوهش دانشکده یا مرکز تحقیقاتی			
5	تحویل طرح به دانشگاه			
6	تصویب طرح در شورای پژوهش دانشگاه			
7	شروع اجرای طرح			
8	تاییدیه کمیته اخلاق			

زمانبندی گردشکار:

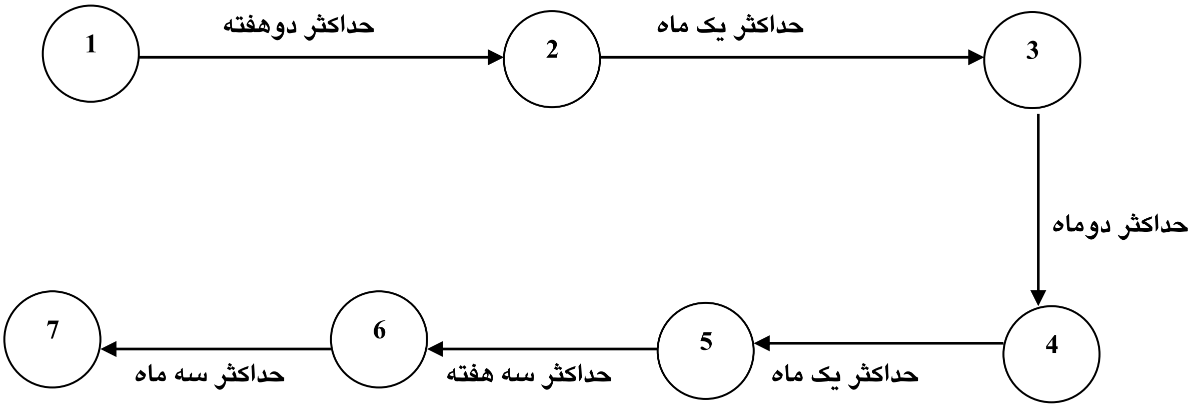

نکات:

1) گردشکار تصویب طبق برنامه راهبردی تحقیقات دانشگاه حداکثر 4 ماه به طول می انجامد .

2) سرپرست طرح می تواند بجای تکمیل فرم عنوان از همان ابتدا شناسنامه طرح را تکمیل و به مراکز تحویل دهد.

3) در صورت عدم مراجعه سرپرست طرح جهت عقد قرارداد پس از سه ماه از تصویب طرح در شورای پژوهش دانشگاه، پژوهشکده و دانشکده ها می توانند پروژه را متوقف نمایند. در هر صورت پس از 3 ماه تأخیر در فاز اجرایی طرح محسوب می شود.

امضای سرپرست

اطلاعات مربوط به سرپرست، ناظر و طرح

نام و نام خانوادگی: دکتر مجید رمضانی **تاریخ تولد :** **شماره شناسنامه :** **نام پدر:**

درجه علمی (دانشگاهی) : **شغل فعلـــی :**

نشانی محل کـار :	تلفن :
نشانی مــنـــزل :	تلفن :

مدرک تحصیلی کارشناسی ارشد و بالاتر خود را در جدول زیر ذکر کنید :

ردیف	مدرک تحصیلی	رشته	دانشگاه	کشور - شهر	سال اخذ
1					
2					
3					

در صورت گذراندن دوره های تخصصی ، جدول زیر را تکمیل نمایید .

ردیف	نام دوره	سازمان / مؤسسه	کشور	مــدت	سال خاتمه
1					

سوابق شرکت سرپرست در طرحهای پژوهشی(در صورت نیاز از برگه های ضمیمه استفاده نمایید)

نوع طرح : بنیادی ☐ کاربردی ■ بنیادی – کاربردی ☐

مدت اجرای طرح: 13 ماه

سازمان مجری : دانشگاه علوم پزشکی بقیه الله

سازمان‌های همکار طرح :

ردیف	نام سازمان	نشانی و تلفن	نوع همکاری
1			

بخش هایی از نیروهای مسلح که نتایج حاصل از انجام طرح مورد نیاز آنها باشد :

مشخصات ناظر(توسط مرکز مربوطه تکمیل گردد):

نام و نام خانوادگی : **تاریخ تولد :** **شماره شناسنامه :** **نام پدر:** **محل صدور :**

آخرین مدرک تحصیلی : **درجه علمی (دانشگاهی) :** **شغل فعلـــی :**

نشانی محل کـار :	تلفن :
نشانی مــنـــزل :	تلفن :

1 – بیان مسأله (با استناد به مطالعات و مستندات موجود به بیان مشکل، گستردگی و وضعیت فعلی آن، اهمیت مسأله و ضرورت پاسخگویی به آن، اشاره‌ای به تاریخچه مشکل، عوامل مؤثر یا مرتبط با مشکل، آثار بهداشتی، اقتصادی، اجتماعی و ... مشکل بپردازید.).

هیپوتیروئیدیسم یا کم کاری تیروئید یک اختلال اندوکرین رایج است که با سطوح پایین هورمون های تیروئیدی در خون تشخیص داده می شود و عمدتا ناشی از کمبود تولید هورمون های تیروئیدی توسط غده تیروئید و یا به طور ثانویه، ناشی از فقدان هورمون محرک تیروئید (TSH) از هیپوفیز قدامی و یا هورمون محرک تیروتروپین (TRH) از هیپوتالاموس می باشد. در ابتدا مرحله ای جبرانی وجود دارد که به واسطه ی افزایش میزان TSH، سطح هورمون های تیروئید در حد طبیعی حفظ می شود. با این حال، ممکن است در بعضی از بیماران علائم ضعیفی ازافسردگی، اختلال حافظه، اضطراب، و درد های بدنی مشخص شود. این مرحله با عنوان هیپوتیروئیدی تحت بالینی یا خفیف شناخته می شود که سطح TSH سرم افزایش یافته (بیش از4mU/L) ولی سطح تیروکسین(T4) سرمی طبیعی است. درمراحل پیشرفته تر، مقادیر T4 آزاد کاهش یافته و مقادیر TSH افزایش بیشتری می یابد و این حالت معمولا همراه با TSH بالاتر از 10 mU/L به هیپوتیروئیدیسم بالینی معروف است (1, 2). با توجه به نقش حیاتی هرمون های تیروئیدی در متابولیسم، تظاهرات بالینی این بیماری شامل خستگی، ضعف، خشکی پوست، عدم تحمل سرما، اشکال در تمرکز و حافظه، یبوست، افزایش وزن و کاهش اشتها، تنگی نفس، صدای خشن، قاعدگی نا منظم، افسردگی، ادم گوده گذار اندام های تحتانی، ریزش مو، تاری دید، اختلال شنوایی، بی حسی، کندی ضربان قلب و هایپرکلسترولمیا می باشد (2). هیپوتیروئیدیسم تحت بالینی یک مشکل رایج با شیوع 8-3٪ است که با افزایش سن افزایش می یابد (3). شیوع آن در زنان بالاتر است اما بعد از دهه 60 زندگی شیوع آن در مردان و زنان نزدیک به 10 درصد می شود (4). بروز کم کاری تیروئید در بین دو سوم جمعیت جهان که در جوامع دریافت کننده ید کافی زندگی می کنند، 1-2٪ گزارش شده است (5). گلد استاندارد درمان هیپوتیروئیدیسم در حال حاضر تجویز لووتیروکسین در دوز تنظیم شده است (6)، هرچند که عوارضی از جمله عوارض نرولوژیک از این دارو گزارش شده (7). از آنجا که خود این بیماری و عوارض متعدد آن بار اقتصادی زیادی بر خانواده و جامعه تحمیل می کند (8)، استفاده از روش های غیر دارویی با عوارض و هزینه کمتر، علاوه بر بیماران می تواند به نفع جامعه و سیستم بهداشتی-درمانی نیز باشد.

پروبیوتیک ها میکروارگانیسم های زنده ای هستند که وقتی به مقدار کافی تجویز شوند، به سلامتی میزبان کمک می کنند، و پره بیوتیک ها نیز غذای این میکروارگانیسم های مفید هستند که به رشد آن ها کمک می کنند (9). ترکیب گونه های پروبیوتیک و پره بیوتیک، اثرات سینرژیستی دارد که به آن سین بیوتیک می گویند. اثرات متقابل بین پرو و پره بیوتیک ها در بدن موجود زنده می‌تواند موجب اصلاح محتوای میکروبیوتا و حفظ تعادل میکروبی روده شود (10).

یکی از اثرات مفید سینبیوتیک ها بهبود افسردگی به واسطه تعدیل و تنظیم سیستم ایمنی و کاهش التهاب است (11). مطالعات حیوانی و انسانی متعددی نشان داده اند که پروبیوتیک ها و سینبیوتیک ها می توانند عوامل التهابی از جمله hs-CRP, TNF-α, IL-6 را کاهش داده و برعکس، عوامل ضد التهابی را افزایش دهند (12-14). عوامل التهابی از طریق اثر بر سیستم عصبی مرکزی می توانند باعث آسیب شده و بیماری های عصبی-روانی از جمله افسردگی و استرس ایجاد کنند (15, 16). از طرفی، مصرف سینبیوتیک ها باعث تغییر مثبت میکروبیوم روده می شوند (17). امروزه تحقیقات علوم اعصاب اهمیت میکروبیوتای روده را در تنظیم سیستم های مغزی اثبات کرده است و مشخص شده که میکروبیوتا بر رفتارهای مربوط به استرس از جمله اضطراب و افسردگی موثر است (18). بنابرین سینبیوتیک ها می توانند اثرات ضد افسردگی داشته باشند. مطالعات اپیدمیولوژیک و بالینی متعددی حاکی از وجود افسردگی در بیماران دچار کم کاری تیروئید هستند (19, 20). هورمون های تیروئیدی انتقال عصبی نورآدرنالین و سروتونین را تحت تأثیر قرار می دهند، که نقشی اساسی در پاتوژنز افسردگی بازی می کنند و اهداف درمان های ضد افسردگی فعلی هستند (21). سروتونین همچنین یک اثر مهاری بر ترشح هورمون TRH دارد که نشان دهنده یک حلقه فیدبکی معیوب است. به عبارت دیگر، کم کاری تیروئید باعث کاهش ترشح سروتونین می شود، و در نبود سروتونین نیز ترشح TRH و به دنبال آن ترشح هرمون های تیروئیدی نیز کاهش می یابد (22). این مساله اهمیت و ضرورت درمان افسردگی در این بیماران را نشان می دهد که می تواند به واسطه مصرف مکمل سینبیوتیک محقق شود.

رشد بیش از حد باکتری های مضر در روده می تواند جذب داروی تیروکسین خوراکی و مواد مغذی از جمله ید را کاهش دهد (23). همچنین شواهد حاکی از آن است که ترکیب میکروبی روده بر متابولیسم یدوتیرونین ها (پیش ساز هرمون های تیروئیدی)، وضعیت ید و سلنیوم (که برای ساخت هرمون های تیروئیدی ضروری اند) و چرخه روده ای-کبدی هرمون های تیروئیدی موثر است (24, 25). مطالعه نریمانی راد و همکاران نشان داد که مصرف پروبیوتیک به مدت یک ماه می تواند سطح هرمون T4 را افزایش و TSH را کاهش دهد (26). Spaggiari و همکاران نیز مشاهده کردند که مکمل یاری با ترکیبی از پروبیوتیک ها می تواند دوز لووتیروکسین مورد نیاز بیماران مبتلا به کم کاری تیروئید را به طور قابل توجهی کاهش دهد (27). فشار خون بالا نیز در بیماران دچار هیپوتیروئیدیسم از شیوع نسبتا بالایی برخوردار است (28) که اثربخشی پروبیوتیک ها در بهبود این عامل خطر نیز در مطالعات مرور سیستماتیک و متاآنالیز اثبات شده (29, 30). از آنجا که سینبیوتیک ها ترکیب پروبیوتیک و پره بیوتیک بوده و به شکل موثرتری می توانند میکروبیوتای روده را اصلاح نمایند، پیش بینی می شود اثرات مثبت بیشتری از مکمل یاری با سینبیوتیک ها بر بهبود پارامترهای هرمونی و فشار خون این بیماران مشاهده شود.

کیفیت زندگی نیز از دیگر عواملی است که تحت تاثیر کم کاری تیروئید قرار می گیرد، چراکه این بیماران به دلیل اختلال در ترشح هرمون های تیروئیدی دچار خستگی، اضافه وزن، خواب آلودگی و اختلالات عصبی-روانی می شوند (31, 32). طبق نتایج تحقیقات پیشین، سطح بالاتر هرمون TSH با وضعیت بدتر کیفیت زندگی مرتبط است (33). همچنین بین سطح سرمی هرمون های تیروئیدی (T3 و T4) و کیفیت زندگی نیز ارتباط معنی داری مشاهده شده است (34). بنابراین می توان امیدوار بود که سینبیوتیک بتواند از طریق اثر بر سطح سرمی هرمون های تیروئیدی و افسردگی، کیفیت زندگی را نیز در بیماران دچار هیپوتیروئیدیسم بهبود بخشد.

بنابراین طبق شواهد به نظر می رسد مکمل یاری با سینبیوتیک بتواند بر بهبود وضعیت بیماران مبتلا به کم کاری تیروئید موثر باشد. با این حال، از آنجا که طبق جستجوی محقق تا کنون تنها یک مداخله با سینبیوتیک در بیماران هیپوتیروئیدی صورت گرفته (35) و برای اثبات اثر این مکمل پژوهش های بیشتری نیاز است، بر آن شدیم تا این کارآزمایی بالینی را طراحی و اجرا نماییم. در این مطالعه تاثیر 10 هفته مکمل یاری با 500 میلی گرم سینبیوتیک بر سطح سرمی هرمون های تیروئیدی، فشار خون، افسردگی، و کیفیت زندگی بیماران هیپوتیروئیدی مورد بررسی قرار خواهد گرفت. در صورت اثبات نتایج مورد انتظار، امید است استفاده از مکمل سینبیوتیک در کنار درمان های رایج هیپوتیروئیدیسم بتواند به کاهش افسردگی، بهبود کیفیت زندگی و کاهش عوارض هرمونی این بیماران کمک کند.

امضای سرپرست

2 – واژه نامه و اصطلاحات فنی:

سینبیوتیک: سین بیوتیک شامل اجزا و مواد غذایی است که شامل پروبیوتیک و پره بیوتیک می باشد که باعث تنظیم بالانس باکتری ها در روده می شود (36).

افسردگی: به حالات خلق افسرده، کاهش علاقه یا لذت، کاهش وزن یا کاهش یا افزایش اشتها، بیخوابی، بیقراری یا کندی حرکتی روانی، خستگی یا از دست دادن انرژی، احساس بی ارزشی یا گناه، مشکل تمرکز یا بی تصمیمی و افکار خودکشی اطلاق می گردد (37).

کیفیت زندگی: برداشت هر شخص از وضعیت سلامتی خود و میزان رضایت از این وضع (38).

فشار خون: فشار مورد نیاز برای جریان خون در رگهای خونی است و در تمام بافتهای بدن انسان گردش می کند. فشار خون از 2 قسمت فشار سیستولیک (SBP) و فشار دیاستولیک (DBP) تشکیل شده است. فشار خون سیستولیک به عنوان فشار در هنگام انقباضات قلب در حالی که فشار خون دیاستولیک به عنوان فشار خون در هنگام آرامش قلب تعریف می شود (39).

تعریف عملی واژه ها:

افسردگی: در این مطالعه ابتلا به افسردگی و شدت آن از طریق پرسشنامه بک ارزیابی خواهد شد.

کیفیت زندگی: در این مطالعه کیفیت زندگی با استفاده از پرسشنامه SF-36 ارزیابی می شود.

فشار خون: در این مطالعه SBP و DBP توسط فشار سنج دیجیتالی برند Omron اندازه گیری خواهد شد.

3 – منابع علمی و سوابق طرح :

1. طالبی و همکاران در سال 2019، تاثیر 8 هفته مکمل یاری با 500 میلی گرم سینبیوتیک را بر 60 بیمار دچار کم کاری تیروئید مورد بررسی قرار دادند. غلظت هورمون TSH، دوز لووتیروکسین و نمره مقیاس شدت خستگی پس از مداخله در گروه سینبیوتیک به طور قابل توجهی کاهش یافت ($P < 0/05$)، در حالی که تفاوت معنی داری در گروه دارونما مشاهده نشد. همچنین سطح FT3 در پایان مداخله در هر دو گروه به طور قابل توجهی افزایش یافت ($P = 0/001$) ولی پس از مداخله بین دو گروه تفاوت معنی دار نبود ($P = 0/259$). فشار خون سیستولیک و فشار خون دیاستولیک نیز در داخل گروه ها یا بین آنها تغییر معنی داری نکرد ($P > 0.05$).
2. Kommers و همکاران در سال 2019 تاثیر مکمل پروبیوتیک بر کیفیت زندگی دانشجویان زن را مورد بررسی قرار دادند. در این کارآزمایی بالینی دو سو کور، پس از یک ماه پایلوت، 32 نفر در گروه مداخله قرار گرفته و به مدت 15 روز مکمل پروبیوتیک دریافت کردند و 32 نفر در همین مدت مکمل دارونما. کیفیت زندگی با استفاده از معیار های ROM III مورد بررسی قرار گرفت. نتایج نشان دهنده بهبود معنی داری کیفیت زندگی در گروه پروبیوتیک نسبت به دارونما بود ($p<0.01$) (40).
3. حقیقت و همکاران در سال 2019 تاثیر مکمل یاری با سینبیوتیک و پروبیوتیک را بر علایم اضطراب و افسردگی در بیماران همودیالیزی بررسی کردند. در این کارآزمایی بالینی سه گروهی، 75 بیمار به سه دسته تقسیم شده و به مدت 12 هفته مکمل سینبیوتیک، پروبیوتیک، یا دارونما مصرف کردند. قبل و بعد از مداخله علایم اضطراب و افسردگی توسط معیارهای بیمارستانی در این بیماران مورد سنجش قرار گرفت. در پایان محققان گزارش کردند که مسزان افسردگی در گروه سینبیوتیک نسبت به گروه های پروبیوتیک و دارونما کاهش بیشتری داشت (به ترتیب $p=0.01$ و $P=0.02$). اما تفاوت معنی داری میان سه گروه از نظر اضطراب مشاهده نشد ($p>0.05$) (41).
4. عاصمی و همکاران در سال 2017 تاثیر مکمل یاری با سینبیوتیک را در بیماران مبتلا به دیابت نوع 2 بررسی کردند. در این کارآزمایی بالینی که به به صورت کراس–اور طراحی شده بود، 62 نفر در گروه مداخله و 62 نفر در گروه دارونما قرار گرفته و به مدت 3 هفته تحت درمان قرار گرفتند. پس از مداخله فشار خون بیماران به طور معنی داری نسبت به قبل از مداخله کاهش معنی داری یافت ($p<0.0001$) اما تفاوت دو گروه در پایان مداخله معنی دار نبود (42).
5. Spaggiari و همکاران در سال 2017 تاثیر دو ماه مکمل یاری با پروبیوتیک را بر بیماران دچار کم کاری تیروئید مصرف کننده لووتیروکسین بررسی کردند. در این کارآزمایی بالینی از گونه های لاکتوباسیلوس و بیفیو باکتر استفاده شد. در پایان مداخله تفاوت معنی داری بین دو گروه از نظر سطح سرمی FT3, FT4, TSH و مارکر بافتی هرمون های تیروئیدی وجود نداشت، اما دوز لووتیروکسین مصرفی در گروه مداخله به طور معنی داری

کاهش، و در گروه کنترل افزایش یافت. محققان نتیجه گیری کردند که پروبیوتیک نمی تواند عملکرد تیروئید را تغییر دهد ولی از نوسانات سطح سرمی هرمون های تیروئیدی جلوگیری می کند (27).

6. نریمانی راد و همکاران در سال 2014 تاثیر 1 ماه مکمل یاری با پروبیوتیک را بر سطخ هرمون های تیروئیدی در ورزشکاران حرفه ای مورد بررسی قرار دادند. پس از مداخله، سطح بالاتری از T4 و سطح پایین تری از TSH در گروه مداخله نسبت به دارونما مشاهده شد ($p<0.01$)،. اما تغییر معنی داری در سطح T3 اتفاق نیفتاد ($p>0.05$). بنابراین محققان نتیجه گرفتند که مکمل یاری با پروبیوتیک ها می تواند بدون اثرات سوء، در تحریک فعالیت غده تیروئید در ورزشکاران موثر باشد (26).

مطالعات مروری و مرور سیستماتیک:

1. Heng Loh و همکاران در مطالعه مرور سیستماتیک خود در سال 2019 به بررسی تاثیر پروبیوتیک ها در بهبود افسردگی و سطح هرمون TSH پرداختند. در این مطالعه اطلاعات مربوط به 12315 نفر آنالیز شد. ابتلا به کم کاری تیروئید خطر افسردگی را 1.7 برابر افراد سالم افزایش می داد (odds ratio 1.72, CI, 1.10 to 2.70; $p = 0.020$). ارتباط معنی داری میان مصرف داری لووتیروکسین و بهبود افسردگی در بیماران هیوتیروئیدی (چه بر اساس پرسشنامه بک و چه بر اساس پرسشنامه همیلتون) مشاهده نشد (43).

2. Liu و همکاران در سال 2019 تاثیر پره بیوتیک و پروبیوتیک بر افسردگی و اضطراب را مورد بررسی قرار دادند. در این مطالعه مرور سیستماتیک 34 مطالعه وارد شدند. پره بیوتیک ها با دارونما از نظر افسردگی ($d = -.08, p =.51$) یا اضطراب ($d =.12, p =.11$) تفاوت نداشتند. پروبیوتیک ها تأثیرات کم اما قابل توجهی در افسردگی ($d = -.24, p <.01$) و اضطراب ($d = -.10, p =.03$) ایجاد کردند. به طور کلی اثرات ضد افسردگی و ضد اضطراب از پروبیوتیک ها مشاهده می شود ولی پره بیوتیک ها چنین اثری نشان ندادند (44).

3. خالصی و همکاران در سال 2014 در یک مرور سیستماتیک و متاآنالیز، تاثیر پروبیوتیک ها بر فشار خون را مورد بررسی قرار دادند. آنها نتیجه گرفتند که مکمل پروبیوتیک می تواند فشار خون سیستولیک را تا 3.58 و فشار خون دیاستولیک را تا 2.83 واحد کاهش دهد. همچنین آنالیز ساب گروپ نشان داد مکمل یاری کمتر از 8 هفته تاثیر معنی داری بر فشار خون نخواهد داشت. همچنین هر چه فشار خون قبل از مداخله بیشتر باشد، تاثیر پروبیوتیک بر کاهش فشار خون بیشتر خواهد بود (45).

جمع بندی:

مطالعات محدودی به بررسی اثر پروبیوتیک و سینبیوتیک در بیماران مبتلا به کم کاری تیروئید پرداخته بودند، که نتایج آن مطالعات اثرات ضد و نقیضی در مورد تاثیر این مکمل ها بر هرمون های تیروئیدی را نشان می داد. اما مداخلات انجام شده در سایر بیماری ها و شرایط حاکی از آن است که پروبیوتیک و سینبیوتیک ها می توانند افسردگی و اضطراب را به طور معنی داری کاهش داده و در بهبود کیفیت زندگی بیماران موثر باشند. تاثیر مکمل های پروبیوتیک و سینبیوتیک بر فشار خون نیز وابسته به مدت زمان مداخله و سطح فشار خون بیماران پیش از مداخله خواهد بود. شبیه ترین مطالعه به تحقیق حاضر، مطالعه طالبی و همکاران می باشد که مکمل سینبیوتیک را به مدت 8 هفته در بیماران هیپوتیروئیدی تجویز نمودند. از آنجا که در این مطالعه تغییری در سطح هرمون های تیروئیدی ایجاد نشد و محققان محدودیت مدت مداخله را به عنوان یکی از دلایل برای این موضوع مطرح کردند، در این مطالعه مدت مداخله را به 10 هفته افزایش دادیم. با توجه به شکاف دانشی موجود در مورد تاثیر مکمل سینبیوتیک بر بیماران دچار کم کاری تیروئید، بر آن شدیم تا این کارآزمایی بالینی را با استفاده از 500 میلی گرم مکمل سینبیوتیک به مدت 10 هفته در بیماران هیپوتیروئیدی اجرا کنیم.

امضای سرپرست

4 - هدف کلی:

تعیین تاثیر مکمل یاری با سینبیوتیک بر هرمون های تیروئیدی، فشار خون، افسردگی و کیفیت زندگی در بیماران دچار کم کاری تیروئید.

5 - اهداف جزیی:

- تعیین و مقایسه ی میانگین سطح سرمی هرمون های TSH و FT4 بین گروه سینبیوتیک و دارونما قبل و در پایان مداخله (پیامد اولیه).
- تعیین و مقایسه میانگین فشار خون سیستولیک و دیاستولیک بین گروه سینبیوتیک و دارونما قبل و در پایان مداخله (پیامد ثانویه).
- تعیین و مقایسه ی میانگین امتیاز پرسشنامه افسردگی (بک) بین گروه سینبیوتیک و دارونما قبل و در پایان مداخله (پیامد ثانویه).
- تعیین و مقایسه ی میانگین امتیاز پرسشنامه کیفیت زندگی (SF-36) و خرده مقیاس های آن بین گروه سینبیوتیک و دارونما قبل و در پایان مداخله (پیامد ثانویه).
- وزن، قد، شاخص توده بدنی، فعالیت بدنی، دریافت کالری کل و درشت مغذی ها، طول مدت بیماری، نوع کم کاری تیروئید، دوز لووتیروکسین، تحصیلات، وضعیت تاهل و شغل نیز جهت کنترل عوامل مخدوشگر مورد اندازه گیری قرار خواهند گرفت.

۶- اهداف کاربردی:

در صورت اثبات فرضیات این پژوهش و پژوهش های آینده، می توان استفاده از مکمل سینبیوتیک جهت بهبود سطح هرمون ها، فشار خون، افسردگی و کیفیت زندگی در کنار داروهای روتین مصرفی بیماران مبتلا به هیپوتیروئیدیسم را پیشنهاد کرد.

۷- فرضیات:

- بین میانگین سطح سرمی هرمون های TSH و FT4 بین گروه سینبیوتیک و دارونما در پایان مداخله تفاوت معنی دار وجود دارد.
- بین میانگین میانگین فشار خون سیستولیک و دیاستولیک بین گروه سینبیوتیک و دارونما قبل و در پایان مداخله تفاوت معنی دار وجود دارد.
- بین میانگین امتیاز پرسشنامه افسردگی (بک) بین گروه سینبیوتیک و دارونما در پایان مداخله تفاوت معنی دار وجود دارد.
- بین میانگین امتیاز پرسشنامه کیفیت زندگی (SF-36) و خرده مقیاس های آن بین گروه سینبیوتیک و دارونما در پایان مداخله تفاوت معنی دار وجود دارد.

۸- سوالات:

-

امضای سرپرست

9 - روش اجرا

1 – 9 – نوع مطالعه :

مطالعه مداخله ای(interventional) و یا کارآزمایی بالینی (clinical trial) تصادفی دو سو کور.

2 – 9 – جامعه مورد مطالعه :

بیماران مبتلا به کم کاری تیروئید مراجعه کننده به بیمارستان بقیه الله

9-3- حجم نمونه و روش محاسبه آن :

برای محاسبه حجم نمونه از مطالعه طالبی و همکاران (35) که یک مطالعه کارآزمایی بالینی با دو گروه موازی بود، استفاده شد. اندازه نمونه طبق رابطه زیر با در نظر گرفتن احتمال خطای نوع اول برابر 0.05 و احتمال خطای نوع دوم برابر 0.80 انجام شد. در مطالعه مذکور تغییرات میانگین ± انحراف معیار گروه پلاسبو و سین بیوتیک در رابطه با سطح هرمون TSH پس از 8 هفته مداخله با سینبیوتیک، به ترتیب 0.08±0.09 و 0.28±0.09 بود. در این مطالعه نیز برای آن که شاهد 0.28 واحد اختلاف سطح سرمی TSH بین دو گروه باشیم، حجم نمونه 24 نفر در هر گروه لازم است. با احتساب ریزش 15٪ ، حجم نمونه بدست آمده در هر گروه 28 نفر خواهد بود.

F tests – Variance: Test of equality (two sample case)

**Analysis:**	A priori: Compute required sample size		
**Input:**	Tail(s)	=	One
	Ratio var1 /var0	=	0.2857
	α err prob	=	0.05
	Power (1−β err prob)	=	0.90
	Allocation ratio N2/N1	=	1
**Output:**	Lower critical F	=	0.4964196
	Upper critical F	=	0.4964196
	Numerator df	=	23
	Denominator df	=	23
	Sample size group 1	=	24
	Sample size group 2	=	24
	Actual power	=	0.9036065

9-4-روش تجزیه و تحلیل داده ها:

نمونه ها به روش نمونه گیری آسان یا در دسترس انتخاب می شوند. برای آنالیز داده ها از آمار توصیفی و تحلیلی استفاده خواهد شد. تعیین نرمال بودن توزیع داده ها با استفاده از آزمون شاپیرو‌ویلک و نمودار های Q-Q و P-P plot انجام می شود. برای توصیف متغیرهای کمی برحسب شرایط از میانگین (انحراف معیار) و یا میانه (چارک اول و چارک سوم) و برای متغیرهای کیفی از گزارش فراوانی(درصد) استفاده خواهد شد. برای مقایسه میانگین پیامدهای کمی بین دو گروه مورد مطالعه از آزمون تی مستقل و یا معادل ناپارامتری آن یعنی آزمون من–ویتنی و برای مقایسه نتایج بین قبل و بعد از مداخله در داخل هر گروه از آزمون تی وابسته یا معادل نا پارامتری آن یعنی آزمون ویل کاکسون استفاده خواهد شد. برای مقایسه فاکتورهای کیفی بین دو گروه نیز آزمون کای دو و یا آزمون دقیق فیشر بکار خواهد رفت. در مقایسه نتایج بین دو گروه مورد مطالعه درصورت نیاز به کنترل روی متغیرهای پایه (baseline) برحسب نوع متغیر، از مدلهای رگرسیونی مناسب استفاده خواهد شد. در صورت از دست دادن شرکت کنندگان و کاهش حجم نمونه به کمتر از 24 نفر در هر گروه، پاور مطالعه احتساب خواهد شد و برای گرفتن نتیجه دقیق تر، آنالیز intention to treat (ITT) انجام خواهد شد. آنالیز داده ها با نرم افزار STATA نسخه 14 انجام خواهد شد. مقدار 0/05 > P-value از نظر آماری معنی دار درنظر گرفته خواهد شد.

9-6نحوه رعایت نکات امنیتی و حفاظتی پروژه:

نمونه گیری با رضایت کامل افراد انجام و کلیه ی نتایج محرمانه تلقی خواهد شد. نام افراد در گزارشات ثبت نخواهد شد و افراد توسط کد های اختصاص داده شده به آنان شناسایی می گردند. خونگیری از بیماران توسط خونگیر ماهر انجام خواهد شد به طوریکه شرکت کنندگان دچار کبود شدگی یا درد نشوند. بابت شرکت در مطالعه و دریافت مکمل ها، افراد متقبل هیچ هزینه ای نخواهند شد. در طول مطالعه به کلیه ی سوالات شرکت کنندگان پاسخ داده خواهد شد و در پایان نتایج پژوهش به اطلاع تمامی شرکت کنندگان خواهد رسید. در هر مرحله ای از مطالعه افراد می توانند از ادامه ی مشارکت در پژوهش انصراف دهند.

9-7-مشکلات و محدودیت ها:

-عدم همکاری بیماران بدلیل احتمال ایجاد عوارض مصرف مکمل

راه حل: توضیح اهداف و مزایای طرح، همچنین از کلیه افراد خواسته می شود که در صورت بروز هر گونه مشکل یا عوارض گوارشی سریعا موضوع را با مجری در میان بگذارند و شماره تلفن مجری در اختیار افراد قرار می گیرد تا در صورت لزوم با وی تماس حاصل نمایند. توصیه های مرتبط با هرگونه مشکل گوارشی احتمالی به شرکت کنندگان ارائه خواهد شد.

- بی نظمی در مصرف مکمل ها توسط شرکت کنندگان

راه حل: پایش و پیگیری تلفنی بیماران به صورت هر دو هفته یکبار.

-محافظه کاری احتمالی برخی از افراد در پاسخگویی به سوالات پرسشنامه های مطالعه

راه حل: جلب اطمینان آنها در مورد بی نام بودن پرسشنامه ها و محرمانه بودن اطلاعات، و مصاحبه با افراد به صورت خصوصی.

-مصرف محصولات پروبیوتیکی توسط افراد شرکت کننده در طول مطالعه

راه حل: جهت پیشگیری از این مشکل قبل از اجرای مداخله آموزش های لازم مبنی بر عدم مصرف کلیه محصولات پروبیوتیک نظیر ماست، پنیر، بستنی و دوغ در طول مطالعه به افراد مورد مطالعه داده می شود.

امضای سرپرست

8-9- روش انجام طرح (شیوه اجرایی مراحل طرح و چگونگی جمع آوری اطلاعات):

پس از تصویب طرح در معاونت پژوهشی و کمیته اخلاق دانشگاه، برای نمونه گیری به بیمارستان بقیه الله مراجعه خواهد شد. از میان بیماران مبتلا به کم کاری تیروئید مراجعه کننده به بیمارستان بقیه الله، افرادی که دارای معیارهای ورود به مطالعه بوده، توضیحات لازم در مورد اهداف و روش اجرای طرح و اطمینان از محفوظ ماندن اطلاعات و اختیاری بودن همکاری را اخذ کرده و در صورت رضایت برای شرکت در مطالعه، پس از امضای رضایتنامه آگاهانه کتبی (پیوست 1) وارد مطالعه خواهند شد. در پژوهش حاضر از روش تصادفی سازی بلوکی با اندازه بلوک 4 تایی استفاده خواهد شد. دنباله های اعداد تصادفی از https://sealedenvelope.com/ بدست خواهند آمد. از نظر مخفی سازی تخصیص، کدهای منحصر به فرد بدست آمده از وبسایت مذکور توسط پرسنل بیمارستانی که از اهداف مطالعه بی اطلاع هستند، روی هر جعبه مکمل نوشته می شود. .. با ورود هر فرد به مطالعه براساس توالی تولید شده، جعبه دارویی که کد مورد نظر در آن ثبت شده است به فرد تخصیص داده خواهد شد و و لذا قبل از انتخاب فرد، کسی از نوع درمانی که وی دریافت خواهد کرد، آگاه نخواهد بود. کدها تا زمان تجزیه و تحلیل داده های نهایی و/یا وضعیت اضطراری نزد پرسنل بیمارستان باقی خواهند ماند. همه شرکت‌کنندگان، محققان، پزشکان، و ارزیاب‌های پیامد نسبت به تخصیص درمان کور خواهند بود. در صورت بروز شرایط اضطراری که دانستن نوع درمان برای مدیریت وضعیت شرکت‌کننده حیاتی است، به کارکنان بیمارستان اجازه داده می‌شود تا کدهای اختصاص داده شده به شرکت‌کننده را اعلام کنند. نوع مداخله دریافتی باید به پرسنل درمان ابلاغ شود، اما نباید در هیچ یک از اسناد مطالعه یا برای بیمار ثبت یا شفاهی فاش شود. رفع کورسازی اورژانسی و علت آن بعدا گزارش خواهد شد.

مکمل سینبیوتیک از شرکت دارویی زیست تخمیر تهیه خواهد شد. کپسول های سینبیوتیک حاوی لاکتوباسیلوس کازئی، لاکتوباسیلوس اسیدوفیلوس، لاکتوباسیلوس رامنوس، لاکتوباسیلوس بولگاریکوس، بیفیدوباکتریوم برو، بیفیدوباکتریوم لانگوم، استرپتوکوکوس ترموفیلوس + فروکتوالیگوساکارید با جمعیت میکروبی 109 CFU/gr هستند. دارونما نیز که در از نظر شکل ظاهری و رنگ کاملا مشابه کپسول های سینبیوتیک هستند (حاوی لاکتوز ، منیزیم استئارات ، تالک ، سیلیکون دی اکساید) از زیست تخمیر تهیه خواهد شد (پیوست 7). خون گیری از بیماران به منظور اندازه گیری سطح سرمی TSH, FT4 صورت خواهد گرفت. سپس در آزمایشگاه به مدت 10 دقیقه با سرعت 3000 دور در دقیقه سانتریفیوژ شده و 1 سی سی سرم بیمار در میکروتیوب ریخته و در فریزر 80- درجه سانتی گراد تا پایان مطالعه نگهداری خواهد شد. پرسشنامه های اطلاعات فردی (پیوست 2)، بک (پیوست 3)، SF-36 (پیوست 4)، پرسشنامه فعالیت بدنی (پیوست 5) و پرسشنامه یادآمد خوراک 24 ساعته (پیوست 6) از طریق مصاحبه برای هر شرکت کننده تکمیل خواهد شد. سپس براساس شماره تصادفی، یک جعبه حاوی 74 عدد کپسول برای مصرف 10 هفته روزانه یک عدد همراه با غذا به هر بیمار داده می شود. طراحی مطالعه به صورت parallel یا موازی خواهد بود. توضیحات لازم در خصوص نحوه مصرف و عوارض احتمالی و راه حل آن ها به بیمار ارائه خواهد شد و از افراد درخواست می شود فعالیت بدنی و رژیم غذایی خود را در طول مطالعه تغییر ندهند. همچنین به بیماران یاد آوری می شود که در طول مدت مداخله از هیچ محصول پروبیوتیکی از جمله ماست یا پنیر پروبیوتیک استفاده نکنند. دوز و پروتکل مصرف داروی لووتیروکسین در طول مطالعه بدون تغییر خواهد ماند. اطلاعات مربوط به عوارض جانبی و سایر اثرات ناخواسته در طی بررسی های هفتگی از طریق رسانه های اجتماعی جمع آوری می شود. همچنین شماره محقق در اختیار شرکت کنندگان قرار می گیرد و از آنها خواسته می شود در صورت بروز عوارض جانبی با آنها تماس بگیرند. در انتهای پژوهش نیز مجددا خونگیری صورت گرفته و پرسشنامه ها تکمیل خواهند شد. محقق هر هفته با شرکت کنندگان، تماس می گیرد و در خصوص مصرف قرص ها پرسش نموده و شرایط پژوهش به آنان یادآوری می شود. همچنین به منظور آگاهی از تبعیت بیماران از مصرف مکمل ها، از بیماران خواسته می شود که در پایان مطالعه جعبه های مکمل را با هر تعداد کپسول باقی مانده به محقق تحویل دهند.

معیارهای ورود به مطالعه:

(1) افراد 25-55 سال مبتلا به کم کاری تیروئید (کامل و تحت بالینی با TSHنرمال و T4 کم یا نرمال) (2) BMI کمتر از 35 (3) تحت درمان با لووتیروکسین (4) تمایل به شرکت در مطالعه

معیارهای عدم ورود:

بارداری یا شیردهی

مصرف سیگار یا الکل

سابقه مصرف مکمل پروبیوتیک یا سینبیوتیک در سه ماه گذشته

مصرف دارو های کاهنده اشتها مانند آنتی بیوتیک ها

استفاده از داروهای آنتی بیوتیک و هر دارویی که با سین بیوتیک ها تداخل دارند

ابتلا به بیماری های گوارشی از جمله زخم معده، اسهال یا یبوست و...

معیارهای خروج:

عدم تمایل به ادامه طرح

تکمیل ناقص پرسشنامه ها (بیش از 40٪ پرسشنامه ناقص باشد)

ابتلا به بیماری های عفونی در طول مطالعه

وقوع بارداری

نیاز به جراحی

تغییر در دوز یا نوع داروی مصرفی

مصرف مواد غذایی حاوی پروبیوتیک در طول مطالعه

اندازه گیری های تن سنجی شامل وزن، قد و نمایه توده بدنی (BMI) برای تمام شرکت کنندگان در ابتدای مطالعه و در پایان آن توسط محقق انجام می شود. اندازه گیری وزن با ترازوی عقربه دار Seca، با حداقل لباس و بدون کفش با دقت 0/1± کیلوگرم و اندازه گیری قد به کمک متر نواری و با دقت 0/5± سانتی متر صورت خواهد گرفت. BMI با استفاده از فرمول وزن بر حسب کیلوگرم تقسیم بر مجذور قد بر حسب متر محاسبه خواهد شد. BMI در محدوده 18.5-24.9 kg/m2 به عنوان طبیعی، کمتر از 18.5 به عنوان لاغری، 29.9– 25 به عنوان اضافه وزن و BMI مساوی 30 و بالاتر به عنوان چاقی در نظر گرفته خواهد شد.

برای ارزیابی سطح فعالیت بدنی از پرسشنامه ی بین المللی فعالیت فیزیکی (IPAQ) استفاده خواهد شد که در سه سطح کم ، متوسط و سنگین طبقه بندی شده است. این پرسشنامه برای افراد 15 تا 69 سال قابل استفاده می باشد و در ایران روایی و پایایی آن بررسی و تایید شده است (46). نوع فعالیت های در نظر گرفته شده در این پرسشنامه عبارتند از : پیاده روی، فعالیت های با شدت متوسط و فعالیت های با شدت زیاد. امتیاز هرکدام از این نوع فعالیت بطور جداگانه و از جمع مدت زمان فعالیت (به دقیقه) با تعداد دفعات تکرار (به روز) محاسبه شده و طبق MET-minutes/week گزارش شدند. بر این اساس سطح فعالیت بدنی سبک، متوسط و شدید برای شرکت کنندگان تعیین خواهد شد. نحوه محاسبه میزان فعالیت بدنی بر حسب MET-minutes/week به شکل زیر محاسبه می گردد:

Walking MET-minutes/week = 3.3 × Walking minutes × Walking days.

Moderate MET-minutes/week = 4.0 × Moderate-intensity activity minutes × Moderate days.

Vigorous MET-minutes/week = 8.0 × Vigorous-intensity activity minutes × Vigorous-intensity days.

Total physical activity MET-minutes/week = Sum of Walking + Moderate + Vigorous METminutes/week scores.

همچنین سطوح فعالیت بدنی به شکل زیر تعریف می شوند :

1. سطح فعالیت بدنی سبک ، عبارت است از کم ترین سطح فعالیت بدنی .
2. سطح فعالیت بدنی متوسط ، عبارت است از یکی از سه تعریف زیر : الف) دست کم 1 دقیقه در روز فعالیت با شدت زیاد برای روز یا بیش تر. ب) دست کم 1 دقیقه در روز پیاده روی ویا فعالیت با شدت متوسط برای روز یا بیش تر. ج) مجموع پیاده روی ، فعالیت با شدت متوسط و فعالیت با شدت زیاد برای روز یا بیش تر بطوری که فعالیت بدنی کل دست کم 600 MET-minutes/week باشد .
3. سطح فعالیت بدنی زیاد، عبارت است از یکی از دو تعریف زیر : الف) فعالیت با شدت زیاد برای روز یا بیش تر به طوری که فعالیت بدنی کل دست کم 1500 MET-minutes/week باشد. ب) مجموع پیاده روی، فعالیت با شدت متوسط و فعالیت با شدت زیاد برای دست کم 7 روز به طوری که فعالیت بدنی کل دست کم 3000 MET-minutes/week باشد.

به منظور کنترل عوامل مخدوشگر از جمله سن، جنس، قد، وزن، BMI، دور کمر، طول دوره بیماری، دوز لووتیروکسین، نوع بیماری (خود ایمنی یا غیر خودایمنی)، تحصیلات، شغل، وضعیت تاهل، فعالیت بدنی، و دریافت های غذایی، این متغیر ها نیز پیش و پس از مداخله در دو گروه اندازه گیری خواهند شد.

به منظور کنترل اثر رژیم غذایی و درشت مغذی ها و ریزمغذی های دریافتی بر پیامدهای مطالعه، دریافت غذایی افراد شرکت کننده در ابتدا و انتهای مداخله مورد ارزیابی قرار می گیرد. به این منظور از پرسشنامه یادآمد غذایی 24 ساعته (24–hour dietary recall) برای 3 روز شامل دو روز عادی و یک روز تعطیل به روش مصاحبه استفاده خواهد شد. مقدار مصرفی هر ماده غذایی بر حسب گرم محاسبه خواهد شد و با استفاده از نرم افزار Nutritionist نسخه 4، متوسط دریافت روزانه کالری، درشت مغذی ها و ریز مغذی ها محاسبه خواهد شد.

کیفیت زندگی بیماران با استفاده از پرسشنامه SF-36 ارزیابی خواهد شد. پرسشنامه دارای 36 عبارت است که 8 حیطه مختلف سلامت را مورد ارزیابی قرار می دهد: سلامت عمومی (5 عبارت)، عملکرد جسمانی (10 عبارت)، محدودیت ایفای نقش به دلایل جسمانی (4 عبارت)، محدودیت ایفای نقش به دلایل عاطفی (3 عبارت)، درد بدنی (2 عبارت)، عملکرد اجتماعی (2 عبارت)، انرژی و شادابی (4 عبارت)، و سلامت روانی (5 عبارت). پایین ترین نمره در این پرسشنامه صفر و بالاترین 100 است. سـئوالات 3 گزینه ای با نمره های 0 ، 50 ، 100 و سئوالات 5 گزینـه ای با نمرات 0 ، 25 ، 50 ، 75 و 100، سوالات 6 گزینـه ای با نمرات 100 ، 80 ، 60 ، 40 ،20 ،0 در نظرگرفتـه شده است که امتیاز بالاتر نشان دهنده عملکـرد بهتـر مـی باشد. پایایی و روایی نسخه فارسی این پرسشنامه توسط منتظری و همکاران (1384) در ایران تائید شده است (0/9–α = 0/7کرونباخ) (38).

افسردگی با استفاده از پرسشنامه بک مورد ارزیابی قرار خواهد گرفت. این پرسشنامه شامل 21 سوال است و جمع نمرات این پرسشنامه از 0 تا 62 می تواند نوسان داشته باشد، و نمره منفی منظور نمی گردد. نبود افسردگی با امتیاز 15 – 1، افسردگی خفیف با امتیاز 31 – 16، افسردگی متوسط با امتیاز 47 – 32، و افسردگی شدید با امتیاز 62 – 48 مشخص خواهد شد. ضریب همبستگی این پرسشنامه توسط بک و همکاران 0.96 تعیین شد (47). روایی و پایایی این پرسشنامه در ایران توسط رجبی و همکاران مورد بررسی و تایید قرار گرفته است (48).

اندازه گیری فشار خون سیستولیک و دیاستولیک پس از استراحت بیمار به مدت 10 دقیقه در دمای معمولی اتاق (27 درجه) توسط فشارسنج دیجیتالی Omron انجام خواهد شد. دو بار اندازه گیری فشار خون از دست چپ بیمار، در حالی که دست با زاویه 90 درجه روی میز قرار گرفته باشد انجام شده، و میانگین دو فشار خون جهت استفاده در آنالیز ها یادداشت خواهد شد.

برای تشخیص نوع کم کاری تیروئید از تست Anti-TPO استفاده خواهد شد. برای ارزیابی سطح سرمیFT4 و TSH، از روش ELIZA و از کیت شرکت پیشتاز طب استفاده خواهد شد.

9 – 9 – جدول متغیرها :

ردیف	متغیر	نقش (مستقل، وابسته، زمینه‌ای، مخدوشگر)	کمی: پیوسته	کمی: گسسته	کیفی: اسمی	کیفی: رتبه ای	تعریف علمی	واحد
1	نوع مداخله	مستقل			*		نوع مداخله ای که هر بیمار بر اساس تخصیص تصادفی دریافت می نماید.	کد درج شده بر روی جعبه کپسول ها
2	TSH	وابسته	*				سطح هرمون TSH موجود در سرم افراد شرکت کننده با استفاده از کیت الایزا	mlU/L
3	FT4	وابسته	*				سطح هرمون FT4 موجود در سرم افراد شرکت کننده با استفاده از کیت الایزا	pmol/L
4	کیفیت زندگی	وابسته	*				کیفیت زندگی، از نظر سلامتی یا سایر جنبه های زندگی اندازه گیری با پرسشنامه SF-36	امتیاز پرسشنامه
5	افسردگی	وابسته	*				خلق افسرده، همراه با علائم جسمی و روحی که باعث مختل شدن فعالیتهای روزمره ی فرد می شود. اندازه گیری با پرسشنامه بک	امتیاز پرسشنامه
6	SBP	وابسته		*			فشار خون در هنگام انقباضات قلب اندازه گیری با فشارسنج دیجیتالی	mmHg
7	DBP	وابسته		*			فشار خون در هنگام آرامش قلب اندازه گیری با فشارسنج دیجیتالی	mmHg
8	سن	زمینه ای	*				تعداد سال های کاملی که از عمر فرد می گذرد، اندازه گیری با پرسش از فرد	سال
9	وزن	مخدوشگر	*				وزن فرد اندازه گیری با ترازوی سکا با دقت 100 گرم	kg
10	قد	زمینه ای	*				قد ایستاده ی فرد اندازه گیری با قدسنج با دقت 0/5 سانتی متر	cm
11	BMI	مخدوشگر	*				شاخص توده ی بدنی حاصل تقسیم وزن به کیلوگرم بر مجذور قد به سانتی متر	-
12	دور کمر (WC)	مخدوشگر	*				اندازه دور کمر فرد در ناحیه دو انگشت پایین تر از ناف اندازه گیری با متر نواری	cm
13	فعالیت بدنی	مخدوشگر				*	میزان فعالیت بدنی فرد در هفته با استفاده از پرسشنامه IPAQ	کم، متوسط، زیاد
14	انرژی دریافتی	مخدوشگر	*				میزان انرژی دریافتی روزانه از غذا با استفاده از پرسشنامه یادآمد خوراک 24 ساعته	Kcal

15	پروتئین دریافتی	مخدوشگر	❊				میزان پروتئین دریافتی روزانه از غذا با استفاده از پرسشنامه یادآمد خوراک 24 ساعته	g
16	چربی دریافتی	مخدوشگر	❊				میزان چربی دریافتی روزانه از غذا با استفاده از پرسشنامه یادآمد خوراک 24 ساعته	g
17	کربوهیدرات دریافتی	مخدوشگر	❊				میزان کربوهیدرات دریافتی روزانه از غذا با استفاده از پرسشنامه یادآمد خوراک 24 ساعته	g
18	فیبر دریافتی	مخدوشگر	❊				میزان فیبر دریافتی روزانه از غذا با استفاده از پرسشنامه یادآمد خوراک 24 ساعته	g
19	میزان تحصیلات	مخدوشگر				❊	درجه ی تحصیلی اخذ شده توسط فرد با پرسش از فرد	زیردیپلم/دیپلم/دانشگاهی
20	شغل	مخدوشگر			❊		کاری که فرد بدان مشغول بوده و در قبال آن مزد دریافت می کند با پرسش از فرد	خانه دار/شاغل
21	وضعیت تاهل	مخدوشگر			❊		مجرد یا متاهل بودن با پرسش از فرد	متأهل/ بیوه/ مجرد/ مطلقه
22	طول مدت بیماری	مخدوشگر		❊			مدت زمان ابتلای فرد به بیماری کم کاری تیروئید با پرسش از فرد	سال
23	Anti-TPO	مخدوشگر		❊			وجود تیروئید پراکسیداز آنتی بادی در خون افراد شرکت کننده با کیت الایزا	کم کاری از نوع خود ایمنی/ غیر خودایمنی

امضای سرپرست

11-2 - هزینه های پشتیبانی

11-2-1- هزینه آزمایشات و خدمات تخصصی

ردیف	موضوع آزمایش یا خدمات تخصصی	مرکز سرویس دهنده	تعداد دفعات	وضعیت تدارکات*	هزینه واحد (ریال)	هزینه کل (ریال)
	آنالیز آماری	کلینیک آمار دانشگاه علوم پزشکی ایران			8000000	8000000
					جمع کل به ریال	8000000

11-2-2- سایر هزینه ها (اجاره ، زیراکس ، فیلم برداری ، اسلاید ، صحافی، ایاب و ذهاب، هدایا و ...)

ردیف	مورد هزینه	تعداد یا مقدار لازم	مدت تخمینی استفاده	وضعیت تدارکات*	هزینه واحد (ریال)	هزینه کل (ریال)
	پرینت پرسشنامه ها	1000 برگ			3000	3000000

جمع کل به ریال	3000000

جمع هزینه‌های پشتیبانی به ریال (آزمایشات و خدمات تخصصی به علاوه سایر هزینه‌ها)	

3-11- هزینه مسافرت‌ها

ردیف	مقصد	علت مسافرت	نوع وسیله نقلیه	تعداد افراد	هزینه اقامت (ریال)	هزینه رفت و آمد(ریال)	هزینه کل (ریال)
						جمع کل به ریال	0

امضای سرپرست

4 - 11 - هزینه وسایل مصرفی

ردیف	نام ماده مصرفی	تعداد یا مقدار لازم	مدت تخمینی استفاده در طرح	وضعیت تدارکات*	قیمت واحد	هزینه کل(ریال)
1	سین بیوتیک (فمی لاکت)	1960 عدد		زیست تخمیر	7000 ریال	13720000 ریال
2	پلاسبو	1960 عدد		زیست تخمیر	2000 ریال	3920000 ریال
					جمع کل به ریال	17640000

5 - 11 - هزینه وسایل سرمایه ای

ردیف	نام دستگاه	تعداد	مدت تخمینی استفاده در طرح	وضعیت تدارکات*	هزینه واحد (ریال)	هزینه کل (ریال)
					جمع کل به ریال	0

*** توضیحات : در ستون وضعیت تدارکات با توجه به موارد زیر عدد مناسب گذارده شود .**

1 – در دانشگاه موجود است و می تواند در اختیار قرار گیرد .

2 – توسط سرپرست با بودجه دانشگاه از داخل کشور خریداری می شود .

3 – توسط سرپرست با بودجه دانشگاه از خارج کشور خریداری می شود .

4 – امکان استفاده از تجهیزات سازمان دیگری موجود است که بسته به مورد هزینه آن محاسبه می شود(نام سازمان قید شود).

امضای سرپرست

6 - 11 - جمع هزینه های طرح

ب ـ سهم سازمان(های) دیگر										الف ـ سهم دانشگاه										تفکیک هزینه های طرح
									**0**										0	1 – هزینه کارکنان
									**0**				8	0	0	0	0	0	0	2 – هزینه‌های پشتیبانی
									**0**							3	0	0	0	3 – هزینه های مسافرت
									**0**			1	7	6	4	0	0	0	0	4– هزینه مواد مصرفی
									**0**										0	5 – هزینه مواد سرمایه ای
									**0**			2	8	6	4	0	0	0	0	6 – جمع هزینه های فوق (بودجه سرپرست)
									**0**											7– هزینهٔ ناظر
									**0**											8– هزینهٔ داوران
									**0**											9– مجموع هزینه‌ها (به تفکیک سهم دانشگاه و سازمان(های) دیگر)
**28640000 ریال**																				10– بودجه کلی طرح (مجموع بند های 9- الف و 9- ب)

تبصره :

هزینـــــه هـــــای دلاری طـــــرح برابـــــر 0 **دلار بـــــوده کـــــه بـــــر اســـــاس مبلـــــغ**

معادل **0** **ریال و در هزینهٔ بند(های)** **0** **منظور گردیده است .**

امضای سرپرست

فرم عملیا ت اجرا یی طرح

شرح دقیق عملیا ت اجرایی	فرد اجراکننده	مکان اجرا	زمان پشنهاد ی (سا عت)	زمان مصوب (ساعت)	توضیحا ت
تهیه پرسشنامه ها و کپسول ها	زهره سجادی هزاوه	–	2 ماه		
ثبت پروتکل در clinicaltrials.gov	زهره سجادی هزاوه	–	1 ماه		
کد گذاری کپسول ها، تمرین مصاحبه و تهیه فایل های اکسل و STATA	زهره سجادی هزاوه	بیمارستان بقیه الله	1 ماه		
تهیه معرفی نامه و نمونه گیری	دکتر مجید رمضانی، مهناز رئیسیان	بیمارستان بقیه الله	6 ماه		
تجزیه و تحلیل داده ها	زهره سجادی هزاوه	کلینیک آمار	2 ماه		
ارائه گزارش نهایی	دکتر مجید رمضانی، زهره سجادی هزاوه	–	1 ماه		
پایش	دکتر مجید رمضانی، زهره سجادی هزاوه، مهناز رئیسیان	–	13 ماه		

امضا، سرپرست

منابع :

1. Jameson JL. Harrison's principles of internal medicine: McGraw-Hill Education; 2018.
2. Gaitonde DY, Rowley KD, Sweeney LB. Hypothyroidism: an update. South African Family Practice. 2012;54(5):384-90.
3. Vanderpump MP. The epidemiology of thyroid disease. British medical bulletin. 2011;99(1).
4. Akter N, Qureshi NK, Ferdous HS. Subclinical Hypothyroidism: A Review on Clinical Consequences and Management Strategies. Journal of Medicine. 2017;18(1):30-6.
5. Taylor PN, Albrecht D, Scholz A, Gutierrez-Buey G, Lazarus JH, Dayan CM, et al. Global epidemiology of hyperthyroidism and hypothyroidism. Nature Reviews Endocrinology. 2018;14(5):301.
6. Krude H, Biebermann H, Schnabel D, Tansek MZ, Theunissen P, Mullis PE, et al. Obesity due to proopiomelanocortin deficiency: three new cases and treatment trials with thyroid hormone and ACTH4–10. The Journal of Clinical Endocrinology & Metabolism. 2003;88(10):4633-40.
7. Bunevičius R, Kažanavičius G, Žalinkevičius R, Prange AJ. Effects of Thyroxine as Compared with Thyroxine plus Triiodothyronine in Patients with Hypothyroidism. New England Journal of Medicine. 1999;340(6):424-9.
8. Donnay S, Balsa JA, Álvarez J, Crespo C, Pérez-Alcántara F, Polanco C. Burden of illness attributable to subclinical hypothyroidism in the Spanish population. Revista clinica espanola. 2013;213(8):363-9.
9. Tsuda H, Miyamoto T. Guidelines for the evaluation of probiotics in food. Report of a joint FAO/WHO working group on drafting guidelines for the evaluation of probiotics in food Guidelines for the evaluation of probiotics in food. Report of a joint FAO/WHO working group on drafting guidelines for the evaluation of probiotics in food, 2002. Food science and technology research. 2010;16(1):87-92.
10. Waitzberg DL, Logullo LC, Bittencourt AF, Torrinhas RS, Shiroma GM, Paulino NP, et al. Effect of synbiotic in constipated adult women–a randomized, double-blind, placebo-controlled study of clinical response. Clinical nutrition. 2013;32(1):27-33.
11. Pandey KR, Naik SR, Vakil BV. Probiotics, prebiotics and synbiotics- a review. Journal of Food Science and Technology. 2015;52(12):7577-87.
12. Cepeda MS, Katz EG, Blacketer C. Microbiome-gut-brain axis: probiotics and their association with depression. The Journal of neuropsychiatry and clinical neurosciences. 2017;29(1):39-44.
13. Mousavi SN, Saboori S, Asbaghi O. Effect of daily probiotic yogurt consumption on inflammation: A systematic review and meta-analysis of randomized Controlled Clinical trials. Obesity Medicine. 2020;18.
14. Simeoli R, Raso GM, Lama A, Pirozzi C, Santoro A, Di Guida F, et al. Preventive and therapeutic effects of Lactobacillus Paracasei B21060-based synbiotic treatment on gut inflammation and barrier integrity in colitic mice. Journal of Nutrition. 2015;145(6):1202-10.
15. Liang S, Wu X, Hu X, Wang T, Jin F. Recognizing depression from the microbiota–gut–brain axis. International journal of molecular sciences. 2018;19(6):1592.
16. Pal A, Barik R. Stress, depression & gut microbiota: The gut-brain axis regulation. International Journal of Pharmaceutical Research. 2020;12(3):35-43.
17. Saulnier DM, Gibson GR, Kolida S. In vitro effects of selected synbiotics on the human faecal microbiota composition. FEMS microbiology ecology. 2008;66(3):516-27.
18. Luna RA, Foster JA. Gut brain axis: diet microbiota interactions and implications for modulation of anxiety and depression. Current opinion in biotechnology. 2015;32:35-41.
19. Dayan CM, Panicker V. Hypothyroidism and depression. European thyroid journal. 2013;2(3):168-79.
20. Samuels MH. Subclinical Hypothyroidism and Depression: Is There a Link? The Journal of clinical endocrinology and metabolism. 2018;103(5):2061-4.

21. Panicker V, Evans J, Bjøro T, Åsvold BO, Dayan CM, Bjerkeset O. A paradoxical difference in relationship between anxiety, depression and thyroid function in subjects on and not on T4: findings from the HUNT study. Clinical endocrinology. 2009;71(4):574-80.
22. Mason GA, Bondy SC, Nemeroff CB, Walker CH, Prange Jr AJ. The effects of thyroid state on beta-adrenergic and serotonergic receptors in rat brain. Psychoneuroendocrinology. 1987;12(4):261-70.
23. Lauritano EC, Bilotta AL, Gabrielli M, Scarpellini E, Lupascu A, Laginestra A, et al. Association between hypothyroidism and small intestinal bacterial overgrowth. The Journal of Clinical Endocrinology & Metabolism. 2007;92(11):4180-4.
24. Virili C, Centanni M. "With a little help from my friends"-the role of microbiota in thyroid hormone metabolism and enterohepatic recycling. Molecular and cellular endocrinology. 2017;458:39-43.
25. Virili C, Centanni M. Does microbiota composition affect thyroid homeostasis? Endocrine. 2015;49(3):583-7.
26. Narimani-Rad M, Mesgari M, Lotfi A. Investigation on thyroid hormones level in probiotic-supplemented trained athletes. 2014.
27. Spaggiari G, Brigante G, De Vincentis S, Cattini U, Roli L, De Santis MC, et al. Probiotics Ingestion Does Not Directly Affect Thyroid Hormonal Parameters in Hypothyroid Patients on Levothyroxine Treatment. Front Endocrinol (Lausanne). 2017;8:316.
28. Walsh JP, Bremner AP, Bulsara MK, O'Leary P, Leedman PJ, Feddema P, et al. Subclinical thyroid dysfunction and blood pressure: a community-based study. Clin Endocrinol (Oxf). 2006;65(4):486-91.
29. Ejtahed HS, Ardeshirlarijani E, Tabatabaei-Malazy O, Hoseini-Tavassol Z, Hasani-Ranjbar S, Soroush AR, et al. Effect of probiotic foods and supplements on blood pressure: a systematic review of meta-analyses studies of controlled trials. Journal of diabetes and metabolic disorders. 2020;19(1):617-23.
30. Dong JY, Szeto IM, Makinen K, Gao Q, Wang J, Qin LQ, et al. Effect of probiotic fermented milk on blood pressure: a meta-analysis of randomised controlled trials. The British journal of nutrition. 2013;110(7):1188-94.
31. Cooper DS, Biondi B. Subclinical thyroid disease. The Lancet. 2012;379(9821):1142-54.
32. Jaeschke R, Guyatt G, Cook D, Harper S, Gerstein H. Spectrum of quality of life impairment in hypothyroidism. Quality of Life Research. 1994;3(5):323-7.
33. Vigário P, Teixeira P, Reuters V, Almeida C, Maia M, Silva M, et al. Perceived health status of women with overt and subclinical hypothyroidism. Medical Principles and Practice. 2009;18(4):317-22.
34. Kelderman-Bolk N, Visser TJ, Tijssen JP, Berghout A. Quality of life in patients with primary hypothyroidism related to BMI. European journal of endocrinology. 2015;173(4):507-15.
35. Talebi S, Karimifar M, Heidari Z, Mohammadi H, Askari G. The effects of synbiotic supplementation on thyroid function and inflammation in hypothyroid patients: A randomized, double-blind, placebo-controlled trial. Complementary Therapies in Medicine. 2020;48:102234.
36. Anandharaj M, Sivasankari B, Parveen Rani R. Effects of probiotics, prebiotics, and synbiotics on hypercholesterolemia: a review. Chinese Journal of Biology. 2014;2014.
37. سیدمحمدی هرپووسکمی. تهران: نشر روان. 1382.
38. Montazeri A, Vahdaninia M. Translation, reliability and validity of Persian standard tool sf-36. Quarterlypayesh. J Iran Inst Health Sci Res. 2005;5(1):45-56.
39. Joyner MJ, Casey DP. Regulation of increased blood flow (hyperemia) to muscles during exercise: a hierarchy of competing physiological needs. Physiological reviews. 2015.
40. Kommers MJ, Silva Rodrigues RA, Miyajima F, Zavala Zavala AA, Ultramari VRLM, Fett WCR, et al. Effects of Probiotic Use on Quality of Life and Physical Activity in Constipated Female University Students: A Randomized, Double-Blind Placebo-Controlled Study. The Journal of Alternative and Complementary Medicine. 2019;25(12):1163-71.
41. Haghighat N, Rajabi S, Mohammadshahi M. Effect of synbiotic and probiotic supplementation on serum brain-derived neurotrophic factor level, depression and anxiety symptoms in hemodialysis patients: a randomized, double-blinded, clinical trial. Nutritional Neuroscience. 2019:1-10.

42. Asemi Z, Aarabi MH, Hajijafari M, Alizadeh S-A, Razzaghi R, Mazoochi M, et al. Effects of Synbiotic Food Consumption on Serum Minerals, Liver Enzymes, and Blood Pressure in Patients with Type 2 Diabetes: A Double-blind Randomized Cross-over Controlled Clinical Trial. Int J Prev Med. 2017;8:43-.
43. Loh HH, Lim LL, Yee A, Loh HS. Association between subclinical hypothyroidism and depression: an updated systematic review and meta-analysis. BMC psychiatry. 2019;19(1):12.
44. Liu RT, Walsh RFL, Sheehan AE. Prebiotics and probiotics for depression and anxiety: A systematic review and meta-analysis of controlled clinical trials. Neuroscience and Biobehavioral Reviews. 2019;102:13-23.
45. Khalesi S, Sun J, Buys N, Jayasinghe R. Effect of probiotics on blood pressure: a systematic review and meta-analysis of randomized, controlled trials. Hypertension. 2014;64(4):897-903.
46. Moghaddam MB, Aghdam FB, Jafarabadi MA, Allahverdipour H, Nikookheslat SD, Safarpour S. The Iranian Version of International Physical Activity Questionnaire (IPAQ) in Iran: content and construct validity, factor structure, internal consistency and stability. World applied sciences journal. 2012;18(8):1073-80.
47. Beck AT, Steer RA, Carbin MG. Psychometric properties of the Beck Depression Inventory: Twenty-five years of evaluation. Clinical psychology review. 1988;8(1):77-100.
48. Gholamreza R, Sona Karjo K. Psychometric Properties of a Persian-Language Version of the Beck Depression Inventory - Second Edition (BDI-II-Persian). Educational Measurement. 2013;3(10):139

بسمه تعالی

مشخصات کلی پژوهش های غیرصنعتی
(فرم اطلاعات و چرخه تصویب)

**1.عنوان: پژوهش:**<br>**تاثیر مکمل یاری با سینبیوتیک بر هرمون های تیروئیدی، فشار خون، افسردگی و کیفیت زندگی در بیماران دچار کم کاری تیروئید.**	**-سفارش دهنده / کاربر: دکتر مجید رمضانی**<br>**- رده مجری:**<br>**- کارفرما:**<br>**- برآورد اعتبار مورد نیاز (میلیون ریال): 28640000**<br>**- مدت اجرا (به ماه): 13 ماه**<br>**سطح پژوهش: فرا سازمانی ☐ سازمانی■ نیرویی☐**

2. تعریف مسئله با بیان مشکل (حداکثر در چهار سطر):

بیماران مبتلا به کم کاری تیروئید علاوه بر کاهش سطح سرمی هرمون های تیروئیدی، از مشکلاتی از جمله پرفشاری خون، افسردگی و افت کیفیت زندگی رنج می برند. یکی از عوامل موثر در درمان این بیماری، اصلاح بار میکروبی روده است که از طریق مسیر گوارش-تیروئید- مغز، می تواند عوارض کم کاری تیروئید را کنترل نماید. از این رو بر آن شدیم تا این کارآزمایی بالینی را طراحی و اجرا نماییم. در این مطالعه تاثیر **10** هفته مکمل یاری با سینبیوتیک بر سطح سرمی هرمون های تیروئیدی، فشار خون، افسردگی، و کیفیت زندگی بیماران هیپوتیروئیدی مورد بررسی قرار خواهد گرفت.

3.اهداف مورد نظر (حداکثر در چهار بند):

تعیین و مقایسه ی میانگین سطح سرمی هرمون های **TSH** و **FT4** و **Anti-TPO** بین گروه سینبیوتیک و دارونما در پایان مداخله و در داخل هر گروه قبل و بعد از مداخله (پیامد اولیه).
تعیین و مقایسه میانگین فشار خون سیستولیک و دیاستولیک بین گروه سینبیوتیک و دارونما در پایان مداخله و در داخل هر گروه قبل و بعد از مداخله (پیامد ثانویه).
تعیین و مقایسه ی میانگین امتیاز پرسشنامه افسردگی (بک(بین گروه سینبیوتیک و دارونما در پایان مداخله و در داخل هر گروه قبل و بعد از مداخله (پیامد ثانویه).
تعیین و مقایسه ی میانگین امتیاز پرسشنامه کیفیت زندگی (**SF-36**) و خرده مقیاس های آن بین گروه سینبیوتیک و دارونما در پایان مداخله و در داخل هر گروه قبل و بعد از مداخله (پیامد ثانویه).

4. ضرورت اجراء:

اثرات متقابل بین پرو و پره بیوتیک ها در بدن موجود زنده می‌تواند موجب اصلاح محتوای میکروبیوتا و حفظ تعادل میکروبی روده شود. شواهد حاکی از آن است که ترکیب میکروبی روده بر متابولیسم هرمون های تیروئیدی موثر است. سینبیوتیک ها همچنین بر افسردگی، کیفیت زندگی و فشار خون اثر گذارند. بنابراین به نظر می رسد مصرف مکمل سینبیوتیک توسط بیماران مبتلا به کم کاری تیروئید منجر به کاهش عوارض بیماری و بهبود گردد. با این حال تا کنون تنها یک مداخله با سینبیوتیک در بیماران هیپوتیروئیدی صورت گرفته و برای اثبات اثر این مکمل پژوهش های بیشتری نیاز است. از این رو بر آن شدیم تا این کارآزمایی بالینی را طراحی و اجرا نماییم.

5. دستاوردهای پژوهش:

در صورت اثبات فرضیات پژوهش، پزشکان می توانند در کنار درمان های رایج، توصیه به مصرف مکمل سینبیوتیک جهت بهبود سطح هرمون ها، فشار خون، افسردگی و کیفیت زندگی به بیماران مبتلا به هیپوتیروئیدیسم ارائه نمایند.

6. چنانچه پژوهش یا پژوهش های مشابه ای اجراء شده یا در دست اجراء می باشد نام ببرید و دلایل پیشنهاد عنوان جدید را تشریح فرمائید:
برای اثبات اثربخشی مکمل سینبیوتیک در بهبود کم کاری تیروئید نیاز به انجام کار آزمایی های بالینی متعدد می باشد. از آنجا که تا کنون تنها یک مطالعه در این زمینه انجام شده است، نیاز است مطالعات بیشتر با آنالیز آماری قوی تر اجرا گردد که در این طرح محقق خواهد شد.

**7. تأیید رده مجری از جهت امکان پذیری و داشتن توان اجرا:**<br>**نام و نام خانوادگی:** دکتر مجید رمضانی<br>**امضاء**	**8. تأیید رده کاربر از حیث داشتن نیاز:**<br>**نام و نام خانوادگی:**<br>**مسئولیت:** **امضاء**
**9. نظر معاونت / نیرو از جهت نیاز و جایگاه آن در دکترین:**<br>**نام و نام خانوادگی:**<br>**مسئولیت:** **امضاء**	**10. نظر معاونت پژوهش ستاد مشترک:**<br>**نام و نام خانوادگی:** **امضاء**
**تاریخ تنظیم:**	**تلفن تماس رده مجری (الزاما درج شود):**

پیوست 1: فرم رضایت آگاهانه شرکت در طرح تحقیقاتی

رضایت نامه شرکت در طرح : "تاثیر مکمل یاری با سینبیوتیک بر هرمون های تیروئیدی، فشار خون، افسردگی و کیفیت زندگی در بیماران دچار کم کاری تیروئید"

آقای/ خانم محترم

بدین وسیله از جنابعالی جهت شرکت در پژوهش فوق الذکر دعوت به عمل میآید. اطلاعات مربوط به این پژوهش در این برگه خدمتتان ارائه شده است و شما برای شرکت یا عدم شرکت در این پژوهش آزاد هستید.

شما مجبور به تصمیم گیری فوری نیستید و برای تصمیم گیری در این باره میتوانید سوالات خود را از تیم پژوهشی بپرسید و با هر فردی که مایل باشید مشورت نمایید. قبل از امضای این رضایت نامه مطمئن شوید که متوجه تمامی اطلاعات این فرم شده اید و به تمام سوالات شما پاسخ داده شده است.

مجری پژوهش

1. من می‌دانم که اهداف این پژوهش عبارتند از:

- تعیین و مقایسه ی میانگین سطح سرمی هرمون های TSH و FT4 و Anti-TPO بین گروه سینبیوتیک و دارونما بین دو گروه قبل و در پایان مداخله (پیامد اولیه).
- تعیین و مقایسه میانگین فشار خون سیستولیک و دیاستولیک بین گروه سینبیوتیک و دارونما بین دو گروه قبل و در پایان مداخله (پیامد ثانویه).
- تعیین و مقایسه ی میانگین امتیاز پرسشنامه افسردگی (بک(بین گروه سینبیوتیک و دارونما بین دو گروه قبل و در پایان مداخله (پیامد ثانویه).
- تعیین و مقایسه ی میانگین امتیاز پرسشنامه کیفیت زندگی (SF-36) و خرده مقیاس های آن بین گروه سینبیوتیک و دارونما بین دو گروه قبل و در پایان مداخله (پیامد ثانویه).

2. من می‌دانم که شرکتم در این پژوهش کاملاً داوطلبانه است و مجبور به شرکت در این پژوهش نیستم.

3. من می دانم که حتی پس از موافقت با شرکتم در پژوهش می توانم هر وقت که بخواهم پس از اطلاع به مجری از پژوهش خارج شوم.

4. نحوه ی همکاری من در این پژوهش به اینصورت است:

شرکت کنندگان مورد مصاحبه قرار گرفته و پس از خون گیری و اندازه گیری فشار خون، پرسشنامه های اطلاعات فردی، فعالیت بدنی، دریافت غذایی، آزمون های افسردگی و کیفیت زندگی را تکمیل خواهند نمود. سپس افراد به مدت 10 هفته روزانه یک عدد کپسول سینبیوتیک یا دارونما همراه با غذا مصرف خواهند کرد. در پایان مطالعه نیز پرسشنامه ها مجددا توسط شرکت کنندگان تکمیل خواهند شد و خونگیری صورت خواهد گرفت.

5. منافع احتمالی شرکت من در این مطالعه به این شرح است:

دریافت اطلاعات در خصوص وضعیت کیفیت زندگی و افسردگی خود و توصیه های تغذیه ای.

6. آسیب‌ها و عوارض احتمالی شرکت من در این مطالعه به این شرح است:

با توجه به اینکه بیماران درمان معمولا خود را در طول مدت مداخله دریافت خواهند کرد، آسیب یا عارضه ای متوجه شرکت کنندگان نخواهد بود. اما ممکن است در اثر مصرف مکمل سینبیوتیک دچار عوارض گوارشی شوند اما هیچ یک از این عوارض جدی و شدید نخواهند بود و با توصیه های ارائه شده به بیماران قابل کنترل خواهند بود.

7. در صورت عدم تمایل به شرکت در مطالعه روش معمول درمانی برای من ارائه خواهد شد که منافع و عوارض آن به این شرح است:

بیماران در طول مدت پژوهش تحت نظر پزشک بوده و درمان معمول خود را ادامه می دهند.

8. من می‌دانم که دست اندر کاران این پژوهش، کلیه اطلاعات مربوط به من را نزد خود به صورت محرمانه نگه داشته و فقط اجازه دارند نتایج کلی و گروهی این پژوهش را بدون ذکر نام و مشخصات اینجانب منتشر کنند.

9. می دانم که کمیته اخلاق در پژوهش با هدف نظارت بر رعایت حقوق شرکت کنندگان در طرح می تواند به اطلاعاتم دسترسی داشته باشد.

10. می دانم که هیچ یک از هزینه های انجام مداخلات پژوهشی به شرح ذیل بر عهده من نخواهد بود.

هزینه ای بابت پرسشنامه ها و آزمون ها از شرکت کنندگان اخذ نخواهد شد.

11. خانم زهره سجادی هزاوه جهت پاسخگویی به اینجانب معرفی شد و به من گفته شد تا هر وقت مشکلی یا سوالی در رابطه با شرکت در پژوهش مذکور پیش آمد با ایشان در میان بگذارم و راهنمایی بخواهم.

آدرس و شماره تلفن ثابت و همراه ایشان به شرح به من ارائه شد:

- **تلفن همراه:** 09121757715
- **تلفن ثابت: 86704610**

12. من می‌دانم که اگر در حین و بعد از انجام پژوهش هر مشکلی اعم از جسمی و روحی به علت شرکت در این پژوهش برای من پیش آمد درمان عوارض و هرینه های آن و غرامت مربوطه بر عهده مجری خواهد بود.

13. من می‌دانم اگر اشکال یا اعتراضی نسبت به دست اندرکاران یا روند پژوهش دارم می‌توانم با کمیته اخلاق در پژوهش دانشگاه علوم پزشکیبقیه الله به آدرس: **شیخ بهایی** تماس گرفته و مشکل خود را به صورت شفاهی یا کتبی مطرح نمایم.

14. این فرم اطلاعات و رضایت آگاهانه در دو نسخه تنظیم شده و پس از امضا یک نسخه در اختیار من و نسخه دیگر در اختیار مجری قرار خواهد گرفت.

اینجانب .... موارد فوق‌الذکر را خواندم و فهمیدم و بر اساس آن رضایت آگاهانه خود را برای شرکت خود در این پژوهش اعلام می‌کنم.<br><br>امضای شرکت کننده

اینجانب .... خود را ملزم به اجرای تعهدات مربوط به مجری در مفاد فوق دانسته و متعهد می‌گردم در تأمین حقوق و ایمنی شرکت کننده در این پژوهش تلاش نمایم.

مهر و امضای مجری پژوهش

پیوست 2: فرم اطلاعات فردی

عنوان طرح : "تاثیر مکمل یاری با سینبیوتیک بر هرمون های تیروئیدی، فشار خون، افسردگی و کیفیت زندگی در بیماران دچار کم کاری تیروئید"

تاریخ : ☐ **ابتدای مطالعه** ☐ **پایان مطالعه** **کد بیمار :**

1- نام و نام خانوادگی:
2- وزن: .... کیلوگرم
3- قد: .... سانتی متر
4- BMI: .... 1- لاغر 2- نرمال 3- اضافه وزن 4- چاقی درجه 1 5- چاقی درجه 2 6- چاقی درجه 3
5- سن: .... سال
6- مدت زمان ابتلا به کم کاری تیروئید: .... سال
7- نوع کم کاری تیروئید: 1- خودایمنی (Anti TPO مثبت) 2- غیر خودایمنی (Anti TPO منفی)
8- تحصیلات: 1- زیر دیپلم 2- دیپلم 3- دانشگاهی
9- وضعیت تاهل: 1-متاهل 2-مجرد 3-مطلقه 4-بیوه
10- شغل: 1- خانه دار 2- شاغل
11- آیا مکمل تغذیه ای یا دارو مصرف می کنید؟ 1-بله 2- خیر
اگر بله، لطفا نام آن را ذکر نمایید: ....
12- فشار خون سیستولیک (SBP): mmHg...............
13- فشار خون دیاستولیک (DBP): mmHg...............
14- اطلاعات تماس: شماره تلفن ثابت:.. شماره تلفن همراه: ....

نام و نام خانوادگی و امضاء تکمیل کننده پرسشنامه:

پیوست 3: پرسشنامه افسردگی بک

عنوان طرح : "تاثیر مکمل یاری با سینبیوتیک بر هرمون های تیروئیدی، فشار خون، افسردگی و کیفیت زندگی در بیماران دچار کم کاری تیروئید"

تاریخ : **ابتدای مطالعه** ☐ **پایان مطالعه** ☐ **کد بیمار :**

افسردگی دارای جلوه های گوناگونی است که افراد به آسانی از تشخیص آن غافل می شوند. این اختلال دارای طیف وسیعی از نشانه ها از قبیل سردرد، یبوست، کم شدن اشتها، کمر درد، یا فقط خستگی مزمن است. این تست به شما در رسیدن به این مهم کمک می کند. در این پرسشنامه 21 سوال مطرح شده و هر سوال از چهار عبارت تشکیل شده است. هر یک از این عبارت ها در هر سوال بیان کننده حالتی از شخص است. شما باید عبارت های هر گروه را به ترتیب و با دقت بخوانید. سپس در هر گروه عبارتی را انتخاب کنید که بهتر از همه، طرز احساس کنونی شما را بیان می کند. یعنی آنچه را که درست هم اکنون احساس می کنید. پس از انتخاب داخل دایره را تیک بزنید. ا گر در هر سوال بیش از یک عبارت را وصف خود می دانید، سعی کنید عبارتی را که ممکن است بیشتر درباره شما صادق باشد را انتخاب کنید. اگر از بین دو یا چند عبارت مربوطه به یک گروه نتوانستید انتخابی را به عمل آورید به حدس و گمان متوسل شوید در هر صورت هیچ سوالی را بدون پاسخ نگذارید. این آزمون مخصوص افراد بالای 16 سال هست.

.1

○ غمگین نیستم

○ غمگین هستم

○ غم دست بردار نیست

○ تحملم را از دست داده ام

.2

○ به آینده امیدوارم

○ به آینده امیدی ندارم

○ احساس می‌کنم آینده امید بخشی در انتظارم نیست

○ کمترین روزنه امیدی ندارم.

.3

○ ناکام نیستم

○ ناکام تر از دیگرانم

○ به زندگی گذشته‌ام که نگاه می‌کنم هرچه می‌بینم شکست و ناکامی است

○ آدم کاملاً شکست خورده‌ای هستم

.4

○ مثل گذشته از زندگیم راضی هستم

○ مثل سابق از زندگی لذت نمی‌برم

○ از زندگی رضایت واقعی ندارم

○ از هر کس و هر چیز که بگویی ناراضی هستم

5.

- احساس تقصیر نمی‌کنم
- گاهی اوقات احساس تقصیر می‌کنم
- اغلب احساس تقصیر می‌کنم
- همیشه احساس تقصیر می‌کنم

6.

- انتظار مجازات ندارم
- احساس می‌کنم ممکن است مجازات شوم
- انتظار مجازات دارم
- احساس می‌کنم دارم مجازات می‌شوم

7.

- از خود راضی هستم
- از خود ناراضی هستم
- از خودم بدم می‌آید
- از خودم متنفرم

8.

- بدتر از سایرین نیستم
- از خودم به خاطر خطاهایم انتقاد می‌کنم
- همیشه خودم را به خاطر خطاهایم سرزنش می‌کنم
- برای هر اتفاق بدی خودم را سرزنش می‌کنم

9.

- هرگز به فکر خودکشی نمی‌افتم
- فکر خودکشی به سرم زده اما اقدامی نکرده‌ام
- به فکر خودکشی هستم
- اگر بتوانم خودکشی می‌کنم

10.

- بیش از حد معمول گریه نمی‌کنم
- بیش از گذشته گریه می‌کنم
- همیشه گریانم
- قبلاً گریه می‌کردم اما حالا با اینکه دلم هم می‌خواهد نمی‌توانم گریه کنم

.11

- کم حوصله‌تر از گذشته نیستم
- کم حوصله‌تر از گذشته هستم
- اغلب کم حوصله هستم
- همیشه کم حوصله هستم

.12

- مثل همیشه مردم را دوست دارم
- به نسبت گذشته کمتر از مردم خوشم می‌آید
- تا حدود زیادی علاقه‌ام را به مردم از دست داده‌ام
- از مردم قطع امید کرده‌ام، به آنها علاقه‌ای ندارم

.13

- مانند گذشته تصمیم می‌گیرم
- کمتر از گذشته تصمیم می‌گیرم
- نسبت به گذشته تصمیم‌گیری برایم دشوارتر شده است
- قدرت تصمیم گیریم را از دست داده‌ام

.14

- جذابیت گذشته‌ها را ندارم
- نگران هستم که جذابیتم را از دست بدهم
- احساس می‌کنم هر روز که می‌گذرد جذابیتم را بیشتر از دست می‌دهم
- زشت هستم

.15

- به خوبی گذشته کار می‌کنم
- به خوبی گذشته کار نمی‌کنم
- برای اینکه کاری بکنم به خودم فشار زیادی می‌آورم
- دستم به هیچ کاری نمی‌رود

.16

- مثل همیشه خوب می‌خوابم
- مثل گذشته خوابم نمی‌برد

- یکی دوساعتی زودتر از معمول از خواب بیدار می‌شوم خوابیدن دوباره برایم مشکل است
- چند ساعت زودتر از معمول از خواب بیدار می‌شوم و دیگر خوابم نمی‌برد

17.

- بیشتر از گذشته خسته نمی‌شوم
- بیش از گذشته خسته می‌شوم
- انجام هر کاری خسته‌ام می‌کند
- از شدت خستگی هیچ کاری از عهده‌ام ساخته نیست

18.

- اشتهایم تغییری نکرده است
- اشتهایم به خوبی گذشته نیست
- اشتهایم خیلی کم شده است
- به هیچ چیز اشتها ندارم

19.

- اخیراً وزن کم نکرده‌ام
- بیش از دو کیلو و نیم وزن کم نکرده‌ام
- بیش از پنج کیلو از وزن بدنم کم شده است
- بیش از هفت کیلو وزن کم کرده‌ام

20.

- بیش از گذشته بیمار نمی‌شوم
- از سر درد و دل درد و یبوست کمی ناراحتم
- به شدت نگران سلامتی خود هستم
- آنقدر نگران سلامتی خود هستم که دستم به هیچ کاری نمی‌رود

21.

- میل جنسی‌ام تغییری نکرده است
- میل جنسی‌ام کمتر شده است
- میل جنسی‌ام خیلی کم شده است
- کمترین میل جنسی در من نیست

پیوست 4: پرسشنامه کیفیت زندگی SF-36

عنوان طرح : "تاثیر مکمل یاری با سینبیوتیک بر هرمون های تیروئیدی، فشار خون، افسردگی و کیفیت زندگی در بیماران دچار کم کاری تیروئید"

تاریخ : ☐ **ابتدای مطالعه** ☐ **پایان مطالعه** **کد بیمار :**

به هر سوال به همان شکلی که توضیح داده شده است، پاسخ دهید. اگر مطمئن نیستید که چگونه به یک سوال پاسخ دهید، لطفا بهترین پاسخ ممکن را انتخاب کنید.

1 -بطور کلی، سلامتی خود را چگونه توصیف می نمایید. (یکی را مشخص نمایید)

1 .عالی 2 .بسیار خوب 3 .خوب 4 .متوسط 5 .بد

2 -در مقایسه با سال گذشته به طور کلی سلامت خود را در حال حاضر چگونه ارزیابی می کنید. (یکی را مشخص نمایید)

1 .بسیار بهتر از سال گذشته است 2.کمی بهتر از سال گذشته است 3 .تقریبا مشابه سال گذشته است

4 .کمی بدتر از سال گذشته است 5 .بسیار بدتر از سال گذشته است

3 -موارد زیر شامل فعالیت هایی است که شما احتمالا طی یک روز عادی انجام می دهید. آیا وضعیت سلامتی شما در حال حاضر این فعالیتها را محدود کرده است؟ اگر چنین است به چه میزان. (از هر ردیف یک عدد را مشخص نمایید)

فعالیت ها	بله، بسیار محدود شده است	بله، کمی محدود شده است	خیر، اصلا محدود نشده است
الف- فعالیتهای سنگین مانند دویدن، بلند کردن اجسام سنگین، شرکت در ورزشهای قدرتی	1	2	3
ب- فعالیتهای متوسط مثل حرکت دادن یک میز، جابجایی جاروبرقی، انجام ورزشهای سبک	1	2	3
ج- بلند کردن یا حمل خواروبار منزل	1	2	3
د- بالا رفتن از چند راه پله	1	2	3
ه- بالا رفتن از یک راه پله	1	2	3
و- دولا شدن، زانو زدن یا خم شدن	1	2	3
ز- راه رفتن برای بیش از یک کیلومتر	1	2	3
ح- راه رفتن برای بیش از چند کوچه	1	2	3
ط- راه رفتن برای بیش از یک کوچه	1	2	3
ی- حمام کردن یا پوشیدن لباس	1	2	3

4 -آیا طی 4 هفته گذشته در کار یا دیگر فعالیتهای روزمره، به علت وضعیت سلامتی جسمانی خود یکی از مشکلات زیر را داشته اید؟ (از هر ردیف یک عدد را مشخص نمایید)

الف- کاهش مدت زمانی که صرف کار یا سایر فعالیتها نموده اید : بله1 خیر 2

ب- به کمتر از آنچه تمایل داشته اید، دست یافته اید: بله 1 خیر2

ج- در انجام کارهایی خاص یا سایر فعالیتها محدودیت داشته اید: بله1 خیر 2

د- در انجام کار یا سایر فعالیتها دچار مشکل شده اید (برای مثال نیازمند تلاش بیشتری بوده اید): بله 1 خیر 2

5 -آیا طی 4 هفته گذشته در کار و یا سایر فعالیتهای روزمره، به علت مشکلات روحی خود یکی از مشکلات زیر را داشته اید؟ (از هر ردیف یک عدد را مشخص نمایید)

الف- کاهش مدت زمانی که صرف کار یا سایر فعالیتها نموده اید. بله1 خیر 2

ب- به کمتر از آنچه تمایل داشته اید دست یافته اید. بله1 خیر 2

ج- کار یا سایر فعالیتهای خود را با دقت معمول انجام نداده اید. بله1 خیر 2

6 -طی 4 هفته گذشته سلامت جسمانی یا مشکلات روحی شما تا چه حدی فعالیتهای معمول اجتماعی شمارا در رابطه با خانواده، دوستان، همسایگان با مردم مختل کرده بود؟ (یکی را مشخص نمائید)

1 .اصلا 2.کمی 3 .تا حدودی 4 .زیاد 5 .خیلی زیاد

7 -طی 4 هفته گذشته چقدر درد داشته اید؟ (یکی را مشخص نمائید)

1 .اصلا 2 .بسیارکم 3 .کم 4 .تا حدودی 5 .شدید 6 .بسیار شدید

8 -طی 4 هفته گذشته درد تا چه حد در کار معمولی و همیشگی شما اختلال ایجاد کرده بود ؟هم کار بیرون از منزل و هم کار منزل. (یکی را مشخص نمائید)

1 .اصلا 2 .کمی 3 .تا حدودی 4 .زیاد 5 .خیلی زیاد

9 -این پرسشها مربوط به احساسات و وضعیت شما طی 4 هفته گذشته است. لطفا برای هر سوال نزدیکترین پاسخ به احساس خود را انتخاب کنید. (از هر ردیف یک عدد را مشخص نمائید)   چه مدتی طی 4 هفته گذشته:

	**همیشه**	**اغلب اوقات**	**بعضی اوقات**	**به ندرت**	**هیچ وقت**
لف- فردی سرحال و سرزنده بوده اید؟					
ب- فردی بسیار عصبی بوده اید؟					
ج- به حدی غمگین بوده اید که هیچ چیز شما را شاد نمی کند؟					
د- احساس آرامش و امنیت داشته اید؟					
ه- خود را پر از انرژی احساس میکرده اید؟					
و- خود را غمگین و افسرده احساس میکرده اید؟					
ز- احساس ضعف بیش از حد میکرده اید؟					
ح- فردی شاد بوده اید؟					
ط- احساس خستگی میکرده اید؟					

10 -طی 4 هفته گذشته، وضعیت جسمانی یا مشکلات روحی چه مدتی فعالیتهای اجتماعی شما را مختل کرده بود؟ مثل دیدار دوستان، بستگان و غیره. (یکی را مشخص نمایید)

1 .تمام اوقات   2 .بیشتر اوقات   3 .بعضی اوقات   4 .بندرت   5 .هیچ وقت

11 -هر کدام از عبارات زیر تا چه حدی در مورد شما درست یا نادرست است؟ (از هر ردیف یک عدد را مشخص نمایید)

	**کاملا درست است**	**تا حدود زیادی درست است**	**نمیدانم**	**تا حدود زیادی نادرست است**	**کاملا نادرست است**
الف- بنظر میرسد که من نسبت به دیگر افراد راحتتر مبتلا به بیماری می شوم					
ب- سلامتی من مثل دیگر افرادی است که می شناسم.					
ج- انتظار دارم که و ضع سلامتی ام بدتر شود.					
د- وضع سلامتی من عالی است.					

پیوست 5: پرسشنامه خلاصه شده IPAQ

عنوان طرح : "تاثیر مکمل یاری با سینبیوتیک بر هرمون های تیروئیدی، فشار خون، افسردگی و کیفیت زندگی در بیماران دچار کم کاری تیروئید"

تاریخ :   ☐**ابتدای مطالعه**   ☐ **پایان مطالعه**   **کد بیمار :**

تمام فعالیت های شدیدی را که طی 7 روز اخیر انجام داده اید مد نظر قرار دهید. منظور فعالیت هایی است که قدرت بدنی زیادی می خواهد و باعث می شود بسیار شدید تر از حالت عادی نفس بکشید.لطفا فقط فعالیت هایی را مد نظر قرار دهید که حداقل به مدت 10 دقیقه به صورت پیوسته انجام داده اید.

1. در طول 7 روز اخیر چند روز آن را فعالیت بدنی شدید مانند بلند کردن اجسام سنگین، حفاری، بیل زدن باغچه، ایروبیک، دوچرخه سواری سریع، فوتبال و دویدن داشته اید؟

.... روز در هفته

..........فعالیت بدنی شدید نداشته ام (مراجعه به سوال 3)

2. معمولا در این روز ها چه مدت زمانی را برای انجام فعالیت های بدنی شدید به صورت پیوسته صرف کرده اید؟

.... دقیقه در روز

فعالیت های بدنی متوسطی را که در طول 7 روز اخیر انجام داده اید مد نظر قرار دهید.منظور فعالیت هایی است که قدرت متوسطی می خواهد و باعث می شود شما کمی تند تر از حالت عادی نفس بکشید. .لطفا فقط فعالیت هایی را مد نظر قرار دهید که حداقل به مدت 10 دقیقه به صورت پیوسته انجام داده اید.

3. در طول 7 روز اخیر چند روز آن فعالیت فیزیکی متوسط مانند حمل بارهای سبک، دوچرخه سواری با سرعت متوسط یا والیبال انجام داده اید؟

.... روز در هفته

.... فعالیت بدنی متوسط نداشته ام (مراجعه به سوال 5)

4. معمولا در این روز ها چه مدت زمانی را برای انجام فعالیت های بدنی متوسط صرف کرده اید؟

.... دقیقه در روز

لطفا مدت زمانی را که در طول 7 روز گذشته به پیاده روی اختصاص داده اید مد نظر قرار دهید.این قسمت پیاده روی در محل کار، در خانه، برای رفتن از جایی به جای دیگر و هر نوع پیاده روی که شما به عنوان تفریح، ورزش، تمرینات جسمانی یا در اوقات فراغت انجام داده اید را شامل می شود.

5. در طول 7 روز اخیر چند روز آن را به مدت حداقل 10 دقیقه به صورت پیوسته پیاده روی کرده اید؟

.... روز در هفته

.... پیاده روی نداشته ام (مراجعه به سوال 7)

6. معمولا در این روزها چه مدت زمانی را برای انجام پیاده روی صرف کرده اید؟

.... دقیقه در روز

آخرین سوال مربوط به اوقاتی است که شما در طول 7 روز اخیر به نشستن اختصاص داده اید.شامل نشستن در محل کار، در خانه، برای انجام تکالیف و در اوقات فراغت می باشد.این زمان نشستن پشت میز، نشستن یا لم دادن هنگام تماشای تلویزیون یا مطالعه و زمانی که برای نشستن با دوستان و فامیل اختصاص داده اید را هم شامل می شود.

7. در طول 7 روز اخیر چه مدت زمانی را در هر روز به نشستن اختصاص داده اید؟

.... دقیقه در روز

..........نمی دانم/مطمئن نیستم

پایان پرسشنامه

از همکاری شما سپاسگزاریم

پیوست 6: پرسشنامه یادآمد خوراک 24 ساعته

عنوان طرح : "تاثیر مکمل یاری با سینبیوتیک بر هرمون های تیروئیدی، فشار خون، افسردگی و کیفیت زندگی در بیماران دچار کم کاری تیروئید"

تاریخ : **ابتدای مطالعه** ☐ **پایان مطالعه** ☐ **کد بیمار :**

وعده ی غذایی	نوع غذا و اجزای تشکیل دهنده ی آن	مقدار مصرف	تبدیل واحد به گرم	کد
صبحانه				
میان وعده ی صبح				
ناهار				
میان وعده بعد از ظهر				
شام				
میان وعده قبل از خواب				

پیوست 8: جداول تو خالی

جدول 1- مشخصات دموگرافیک و تن سنجی در دو گروه مکمل یاری با سینبیوتیک و دارونما

متغیر		گروه		P-Value
		سینبیوتیک (n=28)	دارونما (n=28)	
سن (سال)				
قد (سانتی متر)				
وزن (کیلوگرم)				
BMI				
فعالیت بدنی (MET-minute/week)				
انرژی دریافتی (گرم)				
پروتئین دریافتی(گرم)				
چربی دریافتی (گرم)				
کربوهیدرات دریافتی (گرم)				
فیبر دریافتی (گرم)				
طول مدت ابتلا به بیماری				
نوع کم کاری تیروئید	خودایمنی			
	غیر خود ایمنی			
شغل	خانه دار			
	شاغل			
تحصیلات	زیر دیپلم			
	دیپلم			
	دانشگاهی			
وضعیت تاهل	مجرد			
	متاهل			

جدول 2- پیامدهای مطالعه در دو گروه مکمل یاری با سینبیوتیک و دارونما

		گروه		P-Value[1]	Adjusted P-Value
		گروه سینبیوتیک n=28	گروه دارونما n=28		
**TSH (mlU/L)**	مقادیر پایه				
	مقادیر پس از مداخله				
	Difference				
	P-Value[2]				
**FT4 (pmol/L)**	مقادیر پایه				
	مقادیر پس از مداخله				
	Difference				
	P-Value[2]				
افسردگی	مقادیر پایه				
	مقادیر پس از مداخله				
	Difference				
	P-Value[2]				
کیفیت	مقادیر پایه				

				مقادیر پس از مداخله	**زندگی**
				Difference	
				P-Value[2]	
				مقادیر پایه	**SBP (mmHg)**
				مقادیر پس از مداخله	
				Difference	
				P-Value[2]	
				مقادیر پایه	**DBP (mmHg)**
				مقادیر پس از مداخله	
				Difference	
				P-Value[2]	

1: Independent t-test or Mann-Whitney

2: Wilcoxon or Paired t-test
