# Supplementary material for: The effect of synbiotic supplementation on hypothyroidism: A randomized double-blind placebo controlled clinical trial
Source: PLoS One. 2023 Feb 6;18(2):e0277213. doi: 10.1371/journal.pone.0277213 (PMC9901790; doi:10.1371/journal.pone.0277213)
Supplement: S2 Protocol — (DOCX) [file pone.0277213.s003.docx]

**Title:**

The Effect of Synbiotic Supplementation on Thyroid Hormones, Blood Pressure, Depression and Quality of Life in Hypothyroid Patients: A Randomized Double-Blind Placebo Controlled Clinical Trial

**Trial registration:**

IRCT20210926052583N1

**Protocol version:**

Version 1, October 2021

**Funding:**

Baqiyatallah University of Medical Sciences provides the financial funding for this research and no funding or sponsorship will be received from other institutions. The funder does not have any role in study design; collection, management, analysis, and interpretation of data; writing of the report; or the decision to submit the report for publication.

**Roles and Responsibilities:**

Zohreh Sajadi Hezaveh

Role: conducting the trial, sampling, statistical analysis, interpretation of the results, manuscript preparation.

Shahid Beheshti University of Medical Sciences

Tehran, Iran (Islamic Republic of)

+98 21 2274 8001

[sajadi.z@sbmu.ac.ir](mailto:sajadi.z@sbmu.ac.ir)

Dr Majid Ramezani

Role: design and supervision of the project, manuscript preparation.

Baqiyatallah University of Medical Sciences

Tehran, Iran (Islamic Republic of)

+98 21 81261

[m_ramezani@sina.tums.ac.ir](mailto:m_ramezani@sina.tums.ac.ir)

Mahnaz Reisian

Role: sampling, data collection.

Iran University of Medical Sciences

Tehran, Iran (Islamic Republic of)

+98 911 252 3506

[reisian2012@gmail.com](mailto:reisian2012@gmail.com)

**Introduction**

**Background and rationale**

Hypothyroidism is a common endocrine disorder diagnosed with low levels of thyroid hormones, mainly due to the lack of thyroid hormone production by the thyroid gland or, secondarily, caused by the lack of thyroid stimulating hormone (TSH) from the anterior pituitary gland or thyrotropin-releasing hormone (TRH) from the hypothalamus (1, 2). Due to the vital role of thyroid hormones in metabolism, the clinical manifestations of this disease include fatigue, difficulty in concentration and memory, constipation, weight gain and loss of appetite, depression, slow heart rate and hypercholesterolemia (3). The prevalence of hypothyroidism is higher in women, but after the age of 60, its prevalence in men and women becomes ~10% (4). The incidence of hypothyroidism is reported to be 1-2% among two-thirds of the world's population living in communities with adequate consumption of iodine (5). The gold standard for treatment of hypothyroidism is currently dose-adjusted levothyroxine (6), although side effects such as neurological alterations have been reported from this drug (7). Because of the disease itself and its many complications, a great economic burden is imposed on the family and society (8), hence, the use of non-pharmacological methods with less complications and costs, in addition to the common treatment can be beneficial to the health care system and hypothyroid patients.

Probiotics are living non-pathogenic micro-organisms that, when received in sufficient doses, contribute to the health of the host; and prebiotics are degraded by these beneficial micro-organisms and enhance their growth or activity (9). The combination of probiotics and prebiotics with synergistic effects is called “synbiotic” (10). Modulating gut microbiota by synbiotic supplementation has shown to have beneficial health effects in various disease and conditions (11, 12).

Evidence suggests that there is an association between gut microbiota and thyroid function (13). Overgrowth of harmful bacteria in the gut can reduce the absorption of oral thyroxine and essential micronutrients for the thyroid gland, including iodine, iron, selenium, zinc and copper (14, 15). It is also suggested that the composition of the gut microbiota affects the metabolism of iodothyronines (precursor of thyroid hormones), and the intestinal-hepatic cycle of thyroid hormones (16, 17). Supplementation of probiotics in animal studies have shown contradictory results regarding thyroid hormones (18, 19), however, human experimental studies have mostly reported positive effect of probiotics on thyroid function (20, 21).

High blood pressure (BP) is relatively common in patients with hypothyroidism (22) and the effectiveness of probiotics in eliminating this risk factor has been approved by systematic review and meta-analysis studies (23, 24). Depression, another common complication of hypothyroidism, could be decreased by probiotic and synbiotic supplementation through reducing inflammation, modulating the gut-brain axis, and increasing serotonin level (25-27). It is also expected that synbiotic supplementation can enhance hypothyroid patients’ quality of life by balancing thyroid hormone and reducing depression (28).

Since synbiotics are a combination of probiotics and prebiotics and can more effectively modify the intestinal microbiota, it is expected that more positive effects of supplementation with synbiotics be observed in these patients. However, based on our best knowledge, only one clinical trial has investigated the effect of this nutritional therapy on hypothyroid patients so far (29).

**Objectives**

Primary Objectives:

Comparing the mean changes in serum levels of free thyroxine (FT4) and TSH as representatives of thyroid function, between the synbiotic and placebo groups and within the two groups before and after the intervention.

Secondary Objectives:

Comparing the mean changes in systolic blood pressure (SBP), diastolic blood pressure (DBP), depression, and quality of life between the synbiotic and placebo groups and within the two groups before and after the intervention. Also, other data including age, weight, height, body mass index (BMI), physical activity (PA), dietary energy and macronutrient intake, education, career, marital status, duration of the disease, dose of levothyroxine, and type of hypothyroidism (immune and non-immune-related) will be gathered to be taken into account as confounders.

Hypothesis:

10 weeks of synbiotic supplementation will significantly improve serum levels of thyroid hormones, depression, quality of life, SBP and DBP in hypothyroid patients.

**Trial design**

We designed a double-blinded, randomized placebo-controlled clinical trial among patients with hypothyroidism. This will be a single-centered trial with a parallel design and a convenience sampling method.

**Methods: Participants, interventions, and outcomes**

**Study setting**

The study will be performed at the endocrine clinic of Baqiyatallah Hospital.

**Eligibility criteria**

Inclusion criteria

Women or men (25-55 years old) with subclinical hypothyroidism (elevated TSH level and a normal or low free T4 level) for at least a year, and BMI less than 35 who are being treated by levothyroxine. Voluntary written informed consent for all patients is mandatory before any study related procedures.

Exclusionary criteria

The participants must not have any of the following: lactating or pregnant; History of taking probiotic or synbiotic supplements in the last three months; Smoking or alcohol consumption; Taking appetite suppressants such as antibiotics; Use of antibiotics and any medications that interact with synbiotics; Gastrointestinal diseases such as stomach ulcers, diarrhea or constipation etc. for three months before or during the intervention; unwillingness to continue cooperation, non-compliance during the intervention, or no signed informed consent. Participants will also be excluded in case of providing incomplete questionnaires (more than 40% of the dietary questionnaire is incomplete), catching infectious diseases such as Covid-19 during the study, occurrence of pregnancy, undergoing surgery, occurrence of a change in the dose or type of medication, consumption of foods enriched with probiotics (e.g. probiotic yoghurt) during the study.

**Interventions**

Therapeutic effect of probiotics and synbiotics on the serum level of thyroid hormones have been assessed with different species and doses of bacteria and follow-up duration between 4 to 12 weeks (30, 31). Previous trials have used either different species of Lactobacillus genus (32) or a combination of Lactobacillus and Bifidobacterium (21, 29-31, 33). The microbial load of the supplements varied between 10^7^ to 10^10^ CFU/g. Inulin, fructo-, and/or galacto- oligosaccharide have been used as the prebiotic component of synbiotic (21, 30, 32). We decided to conduct this intervention with synbiotic instead of probiotic, since the synergistic interaction of probiotic and prebiotic can more effectively improve the microbial composition of the gut and maintain the intestinal microbial balance (10). The duration of intervention was decided to be 10 weeks, as the previous trials with less duration of intervention reported no significant effect of synbiotic or probiotics on serum level of thyroid hormones and BP (29, 33). A comprehensive meta-analysis also showed that less than 8 weeks supplementation with probiotics had no significant effect on BP (34). The synbiotic supplement (Familact, 500 mg) that will be used in this study contains Lactobacillus casei, Lactobacillus acidophilus, Lactobacillus rhamnus, Lactobacillus bulgaricus, Bifidobacterium Breve, Bifidobacterium Longum, Streptococcus thermophilus plus fructo-oligosaccharide with microbial population of 10^9^ CFU/g. Placebos contain lactose, magnesium stearate, talc, and silicon dioxide and will be made in the same shape, color, odor and taste as the synbiotic supplements. Zist-takhmir Pharmacutical company will provide the synbiotic and placebo capsules. All participants will receive a capsule (Synbiotic supplement or placebo) to be consumed with lunch, daily. Also, the participants will receive appropriate recommendations regarding diet and PA. The recommendations will include: to follow a hypo-caloric diet; to avoid excessive intake of high-fat products (whole dairy products, poultry fat (the skin on chicken), processed meat, butter, cream, ice cream, and palm oil); to avoid excessive sugar intake (candy, soda, syrups, sugar-loaded desserts, caramels, chocolate); to increase PA by walking or cycling for at least 20 minutes for at least 5 days a week.

The supplements will be delivered to the participants at the first appointment and the supplement boxes will be collected at the end of the intervention by the outcome assessor. To recall the participants of the supplements intake and follow the routine recommendations, we will use telephone or online applications such as WhatsApp or Telegram. If supplements intake is missed at lunch, patients will be advised to take them during the same day. They will also be asked to report any complications or adverse event during the study period. The intervention will be discontinued in case of severe and serious complications following the supplementation.

**Outcomes**

Schedule of outcome assessment is represented in Figure 2. The primary outcomes include serum levels of TSH and fT4. The secondary outcomes are BP, quality of life, and depression. The information on anthropometric indices, PA, and dietary intake will be collected to control the potential confounders and covariates. The primary and secondary outcomes will be conducted at baseline and the end of the study (10th week) for all participants and change from baseline will be reported. Adherence will be checked every week.

**Figure 1**. Flow chart of the study design.

Eligible hypothyroid adults (women/men) with written informed consent will be recruited from Baqiyatallah hospital and will be enrolled in this RCT (n=56)

Randomize to two parallel treatment groups for 10 weeks (70 days):

1) synbiotic supplement (n= 28)

2) placebo (n= 28)

1

**Baseline information:**

Demographic and Anthropometric information, medical history, physical activity (IPAQ), dietary intake (3-day food record). Laboratory investigations of thyroid hormones, SBP & DBP, quality of life (SF-36), depression (Beck questionnaire).

**Follow-up:**

Every week by telephone or through social media (telegram or WhatsApp) for the recall of: 1) taking the supplements, 2) tracking possible complications following supplement intake, and 3) Emphasis on maintaining the routine diet and physical activity.

**Final assessment:**

Demographic and Anthropometric information, medical history, physical activity (IPAQ), dietary intake (3-day food record). Laboratory investigations of thyroid hormones, SBP & DBP, quality of life (SF-36), depression (Beck questionnaire), and pill counts.

Statistical Analysis

*RCT: randomized controlled trial; IPAQ: International Physical Activity Questionnaire; SF- 36: short form-36 questionnaire for quality of life, SBP: Systolic Blood Pressure; DBP: Diastolic Blood Pressure.*

**Participant timeline**

**Figure 2**. Schedule of enrolment, interventions, and assessments.

|  | **STUDY PERIOD** | | | | | |
| --- | --- | --- | --- | --- | --- | --- |
|  | **Enrolment** | **Allocation** | **Post-allocation** | | | **Close-out** |
| **TIMEPOINT**** | ***Day 0*** | ***Day 0*** | ***M1*** | ***M2*** | ***W10*** | ***W10-W11*** |
| **ENROLMENT:** |  | | | | | |
| ***Eligibility screen*** | X |  |  |  |  |  |
| ***Informed consent*** | X |  |  |  |  |  |
| ***Allocation*** |  | X |  |  |  |  |
| **INTERVENTIONS:** |  | | | | | |
| ***Synbiotic group*** |  |  |  |  |  |  |
| ***Placebo group*** |  |  |  |  |  |  |
| **ASSESSMENTS:** |  | | | | | |
| ***Demographic and Anthropometric Data (age, weight, height, WC, BMI, PA, dietary energy and macronutrient intake, education, career, marital status, duration of the disease, dose of levothyroxine, and type of hypothyroidism)*** | X | X |  |  | X |  |
| ***Primary Outcomes (FT4 and TSH)*** | X | X |  |  | X |  |
| ***Secondary Outcomes (SBP, DBP, depression, and quality of life*** | X | X |  |  | X |  |
| ***Participant Adherence*** |  |  |  |  | X |  |
| ***Adverse Events*** |  |  |  |  | X |  |

***List of specific timepoints.*

*W: week; M: month; WC: waist circumstance; BMI: body mass index; PA: physical activity; FT4: thyroxine; TSH: thyroid stimulating hormone; SBP: systolic blood pressure; DBP: diastolic blood pressure.*

**Sample size**

The sample size was calculated with G-power software version 3.1.9.2. To calculate the sample size, the study of Talebi et al. (29) was used, which was an 8-week clinical trial with two parallel groups. The sample size was calculated considering the probability of the first type error equal to 0.05 (α = 0.05, significance), and the probability of the second type error equal to 0.80 (β = 0.2, power). In this study, the mean change ± standard deviation of placebo and synbiotic groups regarding TSH levels after 8 weeks of intervention were 0.08 ± 0.09 mIU/L and 0.028 ± 0.09 mIU/L, respectively. In this study, in order to observe 0.28 mIU/L difference in serum TSH levels between the two groups at the end of the study, a sample size of 24 patients in each group is required. According to an expected 15% loss to follow-up, the final sample size included in each group will be 28 patients.


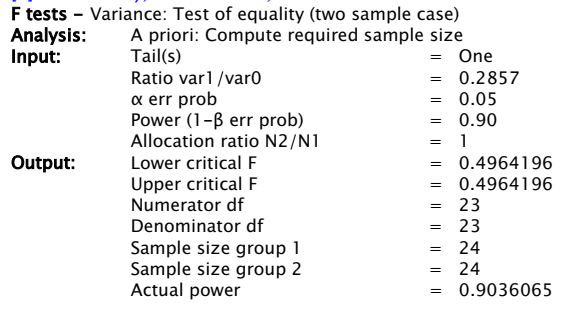


**Recruitment**

The study will be advertised by printed flyers throughout the endocrine clinic of Baqiyatallah Hospital. Flyers will also be handed to practitioners caring for potential participants as well. All advertising material will have the contact number and email address of our research team on it, and interested patients will contact us directly if they wish to hear more about the trial and wish to schedule a telephone screen for eligibility assessment. Afterwards, the eligible and interested individuals will be asked to sign the written informed consent and the first appointment will be booked. During the first appointment, the consented subjects undergo a screening procedure to make sure they meet the inclusion criteria. The eligible subjects will be enrolled in the trial and randomized to appropriate study groups. The participant recruitment period is planned for 8 months and will continue until 56 patients are participated in the study.

**Methods: Assignment of interventions (for controlled trials)**

**Allocation: Sequence generation**

Block randomization method with a block size of 4 will be used in the present study. Random number sequences will be drawn from https://sealedenvelope.com/.

**Allocation concealment mechanism**

In terms of allocation concealment, unique codes derived from the mentioned website will be written on each supplement box by a hospital staff who is not aware of the aims of the study.

**Implementation**

The treatment codes will remain with the staff until the final data analysis and/or an emergency situation. After checking the inclusion and exclusion criteria and baseline measurements, each participant will be given a supplement box.

**Blinding**

All the participants, researchers, practitioners, and outcome assessors will be blinded to the treatment allocation. In the event of an emergency situation where the knowledge of the treatment allocation is critical for further management of the participant, the hospital staff will be given access to unblinding the codes assigned to each participant. The treatment code would be communicated to the medical personnel in charge of the treatment but should not be recorded or verbally disclosed in any of the study documents or to the patient. The emergency unblinding and the reason will be further reported.

**Methods: Data collection, management, and analysis**

**Data collection methods**

Schedule of outcome assessment is represented in Figure 2. The primary outcomes include serum levels of TSH and fT4. The secondary outcomes are BP, quality of life, and depression. The information on anthropometric indices, PA, and dietary intake will be collected to control the potential confounders and covariates. The primary and secondary outcomes will be conducted at baseline and the end of the study (10^th^ week) for all participants. Adherence will be checked every week.

***Laboratory investigations***

All participants will be asked to fast 8-12 hours overnight for blood sample collection (10 ml) at the baseline and the end of study. The samples will be collected into tubes (without anticoagulant and in EDTA in the tubes) and centrifuged at 3000 rpm for 10 min (Hettich D-78532; Tuttlingen, Germany) to obtain serum or plasma. The serum samples (3 ml) will be stored at - 80º c until the end of the trial. Plasma and the remaining serum will be used to determine TSH and fT4 (Pishtazteb Eliza kits, Tehran, Iran).

***Blood Pressure***

BP will be measured at baseline and end of the study by an assessor under the following conditions: After 5 minutes of rest in a quiet place, the participants will be sitting without crossed legs and unsupported back and arms. The BP of the participants will be measured in the left arm at the elbow in a flexed position on the chair by a digital barometer (Omron, Tokyo, Japan). The mean of three readings will be recorded (1-minute interval between them).

***Quality of life***

Short Form Health Survey (SF-36) questionnaire will be used to assess health-related quality of life (HRQOL) in the present study (35). This questionnaire has been translated and validated in the Iranian population (36). SF-36 questionnaire measures 8 domains of health including physical functioning (10 items), role limitations due to physical problems (4 items) and emotional problems (3 items), bodily pain (2 items), general health perceptions (5 items)), vitality (4 items), social functioning (2 items), and perceived mental health (5 items). Also, there is a single item for health transition that provides an indication of perceived change in general health status over a one-year period. Summing the score of the 36 items, an overall score ranging from 0 to 100 will be obtained. The higher the score of SF-36, the higher quality of the participant’s life.

***Depression***

The Beck Depression Inventory II (BDI-II), a self-rating instrument, will be used to assess depression among the participants of the present study (37). An Iranian version of BDI-II has been developed and its reliability and validity has been assessed in the Iranian population (38). The BDI-II consists of 21 sets of statements about depressive symptoms in the last 15 days that are rated on a 0 to 3 ordinal scale, yielding total scores ranging from 0 to 63. The suggested thresholds for levels of severity are as follows: 0-13, minimal/no depression; 14-19, mild depression; 20-28, moderate depression; and 29-63, severe depression.

***Anthropometric indices***

Weight will be measured using a pre-calibrated digital scale (Seca, Germany) to the nearest 0.1 kg. Height will be measured standing up without shoes using a tape measure. Body mass index (BMI) will be calculated by dividing weight (kg) by squared height (m). Waist circumference (WC) will be measured by wrapping the tape measure around the widest part of the stomach, across belly button.

***Physical activity***

The short version of International Physical Activity Questionnaire (IPAQ-SF), which is suitable for adults 15 to 69 years of age, will be used to assess PA in this research (39). IPAQ-SF has acceptable reliability and validity among the Iranian population (40). IPAQ consists of four generic items which reflect on the previous 7 days’ activities (walking, moderate (such as leisure cycling), vigorous activities (such as aerobics), and time spent sitting) and the score within each activity category is reported as minutes per week (min/week) by a Metabolic Equivalent of Task (MET) energy expenditure. The following formula will be used to calculate the PA (MET·min·wk−1): MET level × duration × frequency per week. The results of IPAQ-SF will categorize into 3 levels (vigorous-intensity activity, moderate-intensity activity, low intensity activity) (52). This is a self-report questionnaire and the participants will fill IPAQ-SF.

***Dietary intake***

Dietary intake at the baseline and the end of the study will be recorded using 24-hour dietary recall (completed for 2 weekdays and 1 weekend to account for day-to-day variation). This questionnaire is a structured questionnaire to capture detailed information about all foods and beverages consumed by the respondent in the past 24 hours. The participants will be interviewed by a blinded interviewer to the study allocation. This interviewer encourages the participants to remember the exact type and amount of foods they have eaten by asking proper questions. A blinded expert dietitian to study allocation will review all the completed 24-hour dietary recalls. Dietary intake data will be analyzed using Nutritionist IV software (Version II) to estimate the energy and the amount of macronutrients and micronutrients eaten.

**Data management**

Data entry will be performed along with the sampling. Data will be inserted to an excel sheet by the researcher and will include only the codes, not the names of the participants. This excel file will be stored until the statistical analysis at the end of the trial.

**Statistical methods**

Before statistical analysis, all data will be reviewed in terms of accuracy and completeness. Data will be analyzed using SPSS software (IBM Corp. Released 2016. IBM SPSS Statistics for Windows, Version 24.0. Armonk, NY: IBM Corp). For all analyses, a P value < 0.05 will be considered statistically significant. The normality of data will be checked by the Shapiro-Wilk W Test, P-P and Q-Q plots. All variables will be reported as mean ± SD for before, after the intervention and changes (after minus before). Baseline characteristics of quantitative biochemical and demographic variables will be compared between the two groups by independent sample t-test. The difference between the two groups regarding qualitative variables will be assessed by Chi-square test. Analysis of Covariance (ANCOVA) will be performed to eliminate the effect of confounders. Changes from the base-line to post-intervention within the groups will be illustrated through a paired t-test or Wilcoxon signed-rank test. GraphPad Prism software will be used to draw graphs and illustrate the results (GraphPad Prism version 8.0.0 for Windows, GraphPad Software, San Diego, California USA, [www.graphpad.com](http://www.graphpad.com)).

**Methods: Monitoring**

**Data monitoring**

A data monitoring committee is not considered for this trial as this is a short trial with small sample size.

**Harms**

Information about adverse events and other unintended effects of trial intervention will be collected during the weekly checks through the social media. Participants will also be provided with the number of the researcher and will be asked to make contact in case of adverse events.

**Auditing**

the process of the study will be checked by the ethics committee.

**Ethics and dissemination**

**Research ethics approval**

The study will begin after receiving the ethical approval code from the Medical Ethics Committee of Baqiyatallah University of Medical Sciences. The protocol will then be registered in the Iranian Registry of Clinical Trials (IRCT).

**Protocol Amendments**

Amendments of the protocol will be made after the approval of the Medical Ethics Committee of Baqiyatallah University of Medical Sciences.

**Consent or assent**

The researcher will obtain the written informed consent from all participants prior to participation.

**Confidentiality**

Personal information about potential and enrolled participants will be collected and saved by the researcher in order to protect confidentiality before, during, and after the trial. No one but the researchers will have access to the information. The mentioned information will be made available to the ethics committee upon request.

**Declaration of interests**

No financial or other competing interests exist for principal investigators of this trial.

**Access to data**

The principal investigators (Z.S.H. and M.R.) will have access to the final trial dataset.

**Ancillary and post-trial care**

No ancillary and post-trial care would be provided to the patents unless a persistent complication (e.g. gastro-intestinal) exist following the intervention.

**Dissemination Policy**

The protocol of this study will be available online in two languages of Persian and English through the Iranian Registry of Clinical Trials (IRCT). The results of the present trial will be announced to the participants through the social media. The report of this trial will also be published as an article.

**Appendices**


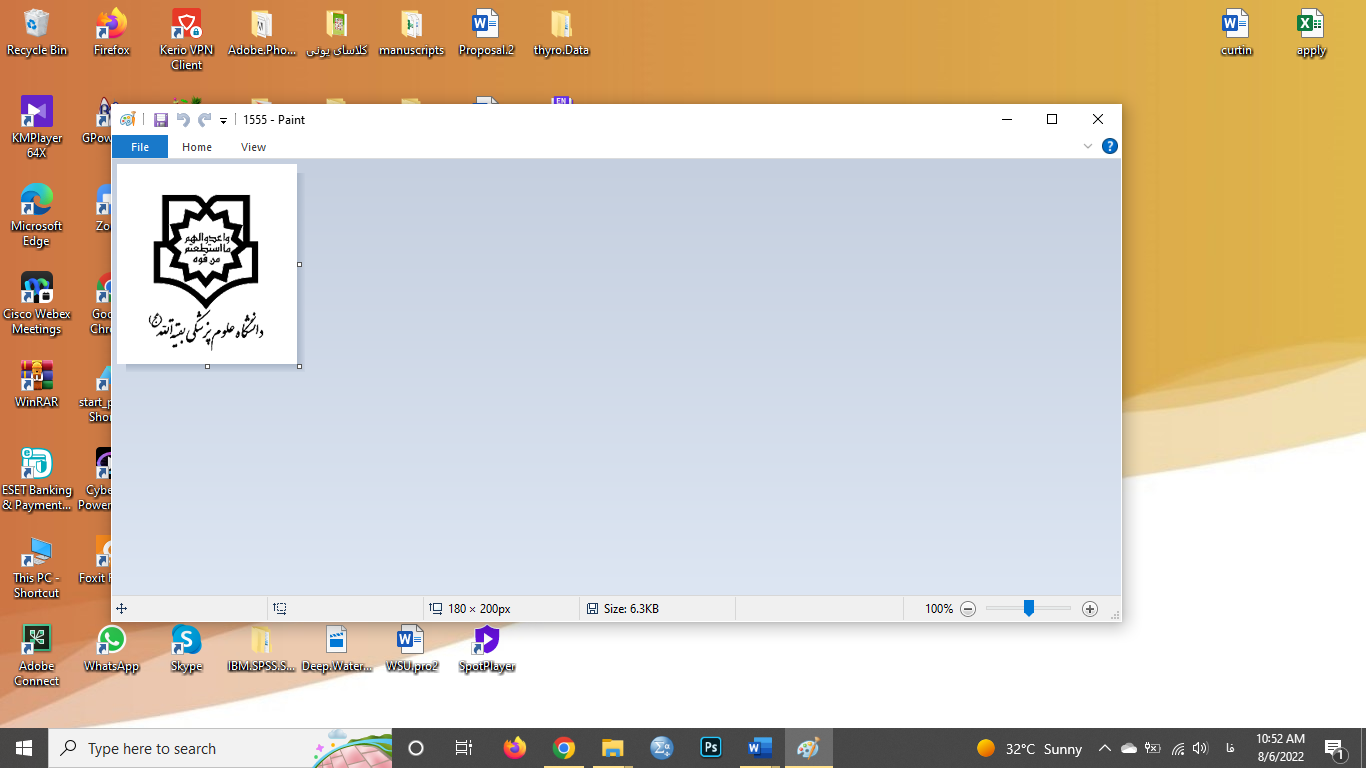
**INFORMED CONSENT FORM**

***School of public Health, Baqiyatallah University of Medical Sciences***

**Title of Project:** The Role of Synbiotic Supplementation in The Treatment of Depression, Quality of Life, Thyroid Hormones, and Blood Pressure in Hypothyroid Patients: A Randomized Double-Blind Placebo Controlled Clinical Trial

**Principal Investigator:** Dr. Majid Ramezani

**Other Investigators**: Zohreh Sajadi Hezaveh

**Participant’s Name:**

**INTRODUCTORY PARAGRAPH**

We invite you to take part in this research study at Baqiyatallah Hospital, which seeks to identify a more effective means of treating beta-thalassemia major.

Taking part in this study is entirely voluntary. We urge you discuss any questions about this study with our staff members. Talk to your family and friends about it and take your time to make your decision. If you decide to participate, you must sign this form to show that you want to take part.

**PURPOSE OF THE RESEARCH**

The purpose of this research study is to obtain information on the effectiveness of synbiotic on hypothyroidism. Approximately 56 people will take part in this research. This research study is being done to find out whether or not synbiotic supplement is effective in controlling the thyroid hormones, depression, quality of life, and blood pressure in hypothyroid patients.

**PROCEDURES**

If you agree to take part in this study, you will be interviewed and you will give us information about your physical activity, dietary intake, and socio-demographic indices (age, smoking status, marital status, etc.). We will take anthropometric measurements including weight and height. Depression and quality of life questionnaires will be obtained. Blood samples will also be taken in the amount of 10cc, so you need to be fast for 10-12 hours. Afterward, a supplement box will be handed to you in which 70 capsules of either synbiotic or placebo exist. A few days after the in-person meeting, we will call you to collect more data on your dietary intake. We will also call you or contact you through social media to make sure you are using the supplements and ask about the possible adverse complication. You are also asked to contact us in case of adverse events.

The same procedure will be repeated after 10 weeks when all the capsules in the supplement box have been consumed. You should bring back the supplement box with you so that we can count the number of the remaining tablets.

**TIME DURATON OF THE PROCEDURES AND STUDY**

If you agree to take part in this study, your involvement will last approximately 10 weeks. You will be asked to return to the clinic after finishing all the tablets in the supplement boxes. Each clinic visit will take approximately 20 minutes. The blood sample will also be taken in less than a minute.

**DISCOMFORTS AND RISKS**

While on the study, you are at risk for the following side effects. Most of them are listed below but they will vary from person to person. These side effects go away after the supplementation is stopped and the side effects are not serious or lasting.

More likely:

- increase in gas and bloating
- constipation
- increased thirst
- Headache

Less likely

- Allergic reaction
- Increased risk of infection

**POTENTIAL BENEFITS**

Synbiotic positively modifies the host’s gut microbial composition, and by improving the gut, it improves the gut-thyroid-brain axis. Many articles reported the protective effects of synbiotic against heart disease, diabetes, cancer, etc. It also has an anti-inflammatory and antioxidative effect, which further improves the overall health status of hypothyroid patients. Preventing neurological diseases and lowering high blood pressure are other benefits of synbiotic. However, there is no guarantee that you will benefit from being in this research.

The results of this research may guide the future treatment of hypothyroidism and medical science may gain further understanding of the possible advantages of this supplement for hypothyroid patients.

**STATEMENT OF CONFIDENTIALITY**

A description of this clinical trial will be available on <https://en.irct.ir/user/trial/58952/>, as required by law. This Web site will not include information that can identify you. At most, the Web site will include a summary of the results. You can search the Web site at any time.

**Privacy and Confidentiality Measures**

Your research records that are reviewed, stored, and analyzed at Baqiyatallah University of Medical Sciences will be kept in a secured area in the nutrition lab. Your blood samples collected for research purposes will be labeled with a code number, and will be stored in -80 ° C freezer. The list that matches your name with the code number will be kept in a locked file in Baqiyatallah University, by the statistician.

If you choose to participate, you are free to withdraw your permission for the use and sharing of your health information and your samples at any time. You must do this in writing. Write to iss Zohreh Sajadi Hezaveh and let him know that you are withdrawing from the research study. His mailing address is zohre_sjd@yahoo.com.

If you withdraw your permission:

- We will no longer use or share medical information about you or your samples for this research study,
- We are unable to take back anything we have already done or any information we have already shared with your permission
- We may continue using and sharing the information obtained prior to your withdrawal if it is necessary for the soundness of the overall research
- We will keep our records of the care that we provided to you as long as the law requires.

**COSTS FOR PARTICIPATION**

Every effort to prevent injury as a result of your participation will be taken. The risks and discomfort due to blood sampling will be minimized, as this procedure will be performed under sterile conditions by highly experienced and qualified people. The blood test is taken while you participants are lying down to avoid light-headedness or fainting during the procedure. However, if complications or injuries occur that are the result of the supplementation, procedure, or test required for this study, the Baqiyatallah University of Medical Sciences will reimburse the standard charges for the treatment of these complications or injuries. The compensation described in this section will be the only form of compensation provided to you for complications or injuries related to this study.

**COMPENSATION FOR PARTICIPATION**

You will not receive any compensation for being in this research study.

**RESEARCH FUNDING**

The institution and investigators are receiving a grant from the Vice Chancellor for research of Baqiyatallah University of Medical Sciences, Tehran, Iran (grant number: 400000020) to support this research.

**VOLUNTARY PARTICIPATION**

Taking part in this research study is voluntary. If you choose to take part in this research, your major responsibilities will include taking the tablets regularly as instructed, and refer to the clinic for the final evaluations. You do not have to participate in this research. If you choose to take part, you have the right to stop at any time. If you decide not to participate or if you decide to stop taking part in the research at a later date, there will be no penalty or loss of benefits to which you are otherwise entitled.

The investigator may take you out of the research study without your permission. Some possible reasons for this are: Pregnancy, surgery, you did not follow the study instructions, or you have serious health issues following the consumption of the supplement. If your participation in the research ends early, you may be asked to visit the investigator for a final visit.

If you will be participating in another clinical trial elsewhere while in this research, you should discuss the procedures and/or treatments with your physician or the investigators. This precaution is intended to protect you from possible side effects from interactions of research drugs, treatments, or testing.

**CONTACT INFORMATION FOR QUESTIONS OR CONCERNS**

You have the right to ask any questions you may have about this research. If you have questions, complaints, or concerns or believe you may have developed an issue related to this research, contact Miss Zohreh Sajadi Hezaveh at +98 912 175 7715.

If you have questions regarding your rights as a research participant or you have concerns or general questions about the research or about your privacy and the use of your personal health information, contact the research subjects protection advocate in the Vice Chancellor for research of Baqiyatallah University of Medical Sciences, Subjects Protection Office at +9821 86 70 4610. You may also call this number if you cannot reach the research team or wish to talk to someone else.

For more information about participation in a research study and about your institutional review board (IRB), a group of people who review the research to protect your rights, please visit the Ethics committee’s Web site at <https://ethics.research.ac.ir/PortalCommitteeEn.php?code=IR.BMSU.REC>. If you do not have access to the Internet, copies of these Federal regulations are available by calling the Ethics committee of the Baqiyatallah University of Medical Sciences at +9821 8145 5618.

**SIGNATURE AND CONSENT/PERMISSION TO BE IN THE RESEARCH**

Before making the decision regarding enrollment in this research, you should have:

- Discussed this study with an investigator
- Reviewed the information in this form
- Had the opportunity to ask any questions you may have.

Your signature below means that you have received this information, have asked the questions you currently have about the research, and have received answers to those questions. You will receive a copy of the signed and dated form to keep for future reference.

**Participant:** By signing this consent form, you indicate that you are voluntarily choosing to take part in this research.

__________________________ __________ ________ ________________________

Signature of Participant Date Time Name

**Participant’s Legally Authorized Representative:** By signing below, you indicate that you give permission for the participant to take part in this research.

__________________________ __________ ________ ________________________

Signature of Participant’s Legally Date Time Name

Authorized Representative

The signature of the participant’s legally authorized representative is required for people unable to give consent for themselves.

Description of the Legally Authorized Representative’s Authority to Act for Participant

**Person Explaining the Research:** Your signature below means that you have explained the research to the participant or participant representative and have answered any questions about the research.

__________________________ __________ ________ __Miss Zoreh Sajadi Hezaveh__

Signature of person who Date Time Name

explained this research

*Only approved investigators for this research may explain the research and obtain informed consent.*

*A witness or witness/translator is required when the participant cannot read the consent document, and it was read or translated.*

1. Gaitonde DY, Rowley KD, Sweeney LB. Hypothyroidism: an update. South African Family Practice. 2012;54(5):384-90.

2. Jameson JL. Harrison's principles of internal medicine: McGraw-Hill Education; 2018.

3. Biondi B, Cooper DS. Thyroid hormone therapy for hypothyroidism. Endocrine. 2019;66(1):18-26.

4. Akter N, Qureshi NK, Ferdous HS. Subclinical Hypothyroidism: A Review on Clinical Consequences and Management Strategies. Journal of Medicine. 2017;18(1):30-6.

5. Taylor PN, Albrecht D, Scholz A, Gutierrez-Buey G, Lazarus JH, Dayan CM, et al. Global epidemiology of hyperthyroidism and hypothyroidism. Nature Reviews Endocrinology. 2018;14(5):301.

6. Krude H, Biebermann H, Schnabel D, Tansek MZ, Theunissen P, Mullis PE, et al. Obesity due to proopiomelanocortin deficiency: three new cases and treatment trials with thyroid hormone and ACTH4–10. The Journal of Clinical Endocrinology & Metabolism. 2003;88(10):4633-40.

7. Bunevičius R, Kažanavičius G, Žalinkevičius R, Prange AJ. Effects of Thyroxine as Compared with Thyroxine plus Triiodothyronine in Patients with Hypothyroidism. New England Journal of Medicine. 1999;340(6):424-9.

8. Donnay S, Balsa JA, Álvarez J, Crespo C, Pérez-Alcántara F, Polanco C. Burden of illness attributable to subclinical hypothyroidism in the Spanish population. Revista clinica espanola. 2013;213(8):363-9.

9. Duca FA, Lam T. Gut microbiota, nutrient sensing and energy balance. Diabetes, Obesity and Metabolism. 2014;16(S1):68-76.

10. Waitzberg DL, Logullo LC, Bittencourt AF, Torrinhas RS, Shiroma GM, Paulino NP, et al. Effect of synbiotic in constipated adult women–a randomized, double-blind, placebo-controlled study of clinical response. Clinical nutrition. 2013;32(1):27-33.

11. Hadi A, Alizadeh K, Hajianfar H, Mohammadi H, Miraghajani M. Efficacy of synbiotic supplementation in obesity treatment: a systematic review and meta-analysis of clinical trials. Critical reviews in food science and nutrition. 2020;60(4):584-96.

12. Tabrizi R, Ostadmohammadi V, Lankarani KB, Akbari M, Akbari H, Vakili S, et al. The effects of probiotic and synbiotic supplementation on inflammatory markers among patients with diabetes: A systematic review and meta-analysis of randomized controlled trials. European journal of pharmacology. 2019;852:254-64.

13. Knezevic J, Starchl C, Tmava Berisha A, Amrein K. Thyroid-Gut-Axis: How Does the Microbiota Influence Thyroid Function? Nutrients. 2020;12(6):1769.

14. Lauritano EC, Bilotta AL, Gabrielli M, Scarpellini E, Lupascu A, Laginestra A, et al. Association between hypothyroidism and small intestinal bacterial overgrowth. The Journal of Clinical Endocrinology & Metabolism. 2007;92(11):4180-4.

15. Fröhlich E, Wahl R. Microbiota and thyroid interaction in health and disease. Trends in Endocrinology & Metabolism. 2019;30(8):479-90.

16. Virili C, Centanni M. Does microbiota composition affect thyroid homeostasis? Endocrine. 2015;49(3):583-7.

17. Virili C, Centanni M. “With a little help from my friends”-the role of microbiota in thyroid hormone metabolism and enterohepatic recycling. Molecular and cellular endocrinology. 2017;458:39-43.

18. Chotinsky D, Mihaylov R. Effect of probiotics and avotan on the level of thyroid hormones in the blood plasma of broiler chickens. Bulg J Agric Sci. 2013;19:817-21.

19. Zhou J, Gill H. Immunostimulatory probiotic Lactobacillus rhamnosus HN001 and Bifidobacterium lactis HN019 do not induce pathological inflammation in mouse model of experimental autoimmune thyroiditis. International journal of food microbiology. 2005;103(1):97-104.

20. Spaggiari G, Brigante G, De Vincentis S, Cattini U, Roli L, De Santis MC, et al. Probiotics ingestion does not directly affect thyroid hormonal parameters in hypothyroid patients on levothyroxine treatment. Frontiers in Endocrinology. 2017;8:316.

21. Narimani-Rad M, Mesgari M, Lotfi A. Investigation on thyroid hormones level in probiotic-supplemented trained athletes. 2014.

22. Walsh JP, Bremner AP, Bulsara MK, O'Leary P, Leedman PJ, Feddema P, et al. Subclinical thyroid dysfunction and blood pressure: a community-based study. Clin Endocrinol (Oxf). 2006;65(4):486-91.

23. Ejtahed HS, Ardeshirlarijani E, Tabatabaei-Malazy O, Hoseini-Tavassol Z, Hasani-Ranjbar S, Soroush AR, et al. Effect of probiotic foods and supplements on blood pressure: a systematic review of meta-analyses studies of controlled trials. Journal of diabetes and metabolic disorders. 2020;19(1):617-23.

24. Dong JY, Szeto IM, Makinen K, Gao Q, Wang J, Qin LQ, et al. Effect of probiotic fermented milk on blood pressure: a meta-analysis of randomised controlled trials. The British journal of nutrition. 2013;110(7):1188-94.

25. Cepeda MS, Katz EG, Blacketer C. Microbiome-gut-brain axis: probiotics and their association with depression. The Journal of neuropsychiatry and clinical neurosciences. 2017;29(1):39-44.

26. Luna RA, Foster JA. Gut brain axis: diet microbiota interactions and implications for modulation of anxiety and depression. Current opinion in biotechnology. 2015;32:35-41.

27. Mousavi SN, Saboori S, Asbaghi O. Effect of daily probiotic yogurt consumption on inflammation: A systematic review and meta-analysis of randomized Controlled Clinical trials. Obesity Medicine. 2020:100221.

28. Kelderman-Bolk N, Visser TJ, Tijssen JP, Berghout A. Quality of life in patients with primary hypothyroidism related to BMI. European journal of endocrinology. 2015;173(4):507-15.

29. Talebi S, Karimifar M, Heidari Z, Mohammadi H, Askari G. The effects of synbiotic supplementation on thyroid function and inflammation in hypothyroid patients: A randomized, double‑blind, placebo‑controlled trial. Complementary Therapies in Medicine. 2020;48:102234.

30. Haghighat N, Rajabi S, Mohammadshahi M. Effect of synbiotic and probiotic supplementation on serum brain-derived neurotrophic factor level, depression and anxiety symptoms in hemodialysis patients: a randomized, double-blinded, clinical trial. Nutritional Neuroscience. 2019:1-10.

31. Kommers MJ, Silva Rodrigues RA, Miyajima F, Zavala Zavala AA, Ultramari VRLM, Fett WCR, et al. Effects of Probiotic Use on Quality of Life and Physical Activity in Constipated Female University Students: A Randomized, Double-Blind Placebo-Controlled Study. The Journal of Alternative and Complementary Medicine. 2019;25(12):1163-71.

32. Asemi Z, Aarabi MH, Hajijafari M, Alizadeh S-A, Razzaghi R, Mazoochi M, et al. Effects of Synbiotic Food Consumption on Serum Minerals, Liver Enzymes, and Blood Pressure in Patients with Type 2 Diabetes: A Double-blind Randomized Cross-over Controlled Clinical Trial. Int J Prev Med. 2017;8:43-.

33. Spaggiari G, Brigante G, De Vincentis S, Cattini U, Roli L, De Santis MC, et al. Probiotics Ingestion Does Not Directly Affect Thyroid Hormonal Parameters in Hypothyroid Patients on Levothyroxine Treatment. Front Endocrinol (Lausanne). 2017;8:316.

34. Khalesi S, Sun J, Buys N, Jayasinghe R. Effect of probiotics on blood pressure: a systematic review and meta-analysis of randomized, controlled trials. Hypertension. 2014;64(4):897-903.

35. Bowden A, Fox-Rushby JA. A systematic and critical review of the process of translation and adaptation of generic health-related quality of life measures in Africa, Asia, Eastern Europe, the Middle East, South America. Social science & medicine. 2003;57(7):1289-306.

36. Montazeri A, Goshtasebi A, Vahdaninia M, Gandek B. The Short Form Health Survey (SF-36): translation and validation study of the Iranian version. Quality of life research. 2005;14(3):875-82.

37. Jackson-Koku G. Beck depression inventory. Occupational Medicine. 2016;66(2):174-5.

38. Gholamreza R, Sona Karjo K. Psychometric Properties of a Persian-Language Version of the Beck Depression Inventory - Second Edition (BDI-II-Persian). Educational Measurement. 2013;3(10):139.

39. Craig CL, Marshall AL, Sjöström M, Bauman AE, Booth ML, Ainsworth BE, et al. International physical activity questionnaire: 12-country reliability and validity. Medicine & science in sports & exercise. 2003;35(8):1381-95.

40. Vasheghani-Farahani A, Tahmasbi M, Asheri H, Ashraf H, Nedjat S, Kordi R. The Persian, last 7-day, long form of the International Physical Activity Questionnaire: translation and validation study. Asian journal of sports medicine. 2011;2(2):106.
