# Supplementary material for: The effect of synbiotic supplementation on hypothyroidism: A randomized double-blind placebo controlled clinical trial
Source: PLoS One. 2023 Feb 6;18(2):e0277213. doi: 10.1371/journal.pone.0277213 (PMC9901790; doi:10.1371/journal.pone.0277213)
Supplement: S1 Table — (DOCX) [file pone.0277213.s004.docx]

**Supplementary Table 1:** Number of missing data at baseline

|  | **Number of Missing data** | |
| --- | --- | --- |
| **Variable** | **Symbiotic** | **Placebo** |
| **Age (year)** | 0 | 0 |
| **Weight (Kg)** | 1 | 0 |
| **Height (cm)** | 1 | 0 |
| **BMI (kg/cm^2^)** | 1 | 0 |
| **Levothyroxine dose (µg)** | 1 | 3 |
| **Duration of Hypothyroidism (year)** | 0 | 0 |
| **Physical activity before** | 0 | 0 |
| **Physical activity after** | 2 | 3 |
| **Calorie (Kcal)** | 1 | 0 |
| **Calorie after (Kcal)** | 2 | 3 |
| **Carbohydrate before (gr)** | 1 | 0 |
| **Carbohydrate after (gr)** | 2 | 3 |
| **Protein before (gr)** | 1 | 0 |
| **Protein after (gr)** | 2 | 3 |
| **Fat before (gr)** | 1 | 0 |
| **Fat after (gr)** | 2 | 3 |
| **Fiber before (gr)** | 1 | 0 |
| **Fiber after (gr)** | 2 | 3 |
| **Sex** | 0 | 0 |
| **Type of Hypothyroidism** | 4 | 7 |
| **Education** | 0 | 0 |
| **Marita Status** | 0 | 0 |
| **Vitamin-mineral supplement intake** | 0 | 0 |
