# Supplementary material for: The effect of synbiotic supplementation on hypothyroidism: A randomized double-blind placebo controlled clinical trial
Source: PLoS One. 2023 Feb 6;18(2):e0277213. doi: 10.1371/journal.pone.0277213 (PMC9901790; doi:10.1371/journal.pone.0277213)
Supplement: S2 Table — 1: within group comparison, paired t-test, 2: between group comparison, independent t-test, 3: two-way ANCOVA analysis with adjustment for duration of hypothyroidism, age, levothyroxine dose, and physical activity. (DOCX) [file pone.0277213.s005.docx]

**Supplementary Table 2:** ITT analysis of the outcomes.

| **P-value^1^** | **Change** | **After** | | **Before** | | |  | **Variable** |  |
| --- | --- | --- | --- | --- | --- | --- | --- | --- | --- |
| 0.01 | 2.40 ± 4.58 | 11.91 ± 3.61 | | 9.51 ± 2.86 | | | **Symbiotic** | **Free T4 (µg/dl)** | **Thyroid Hormones** |
| 0.01 | 2.97 ± 5.50 | 13.55 ± 6.37 | | 10.57 ± 3.55 | | | **Placebo** |  |  |
|  | 0.67 | 0.24 | | 0.22 | | | **P-value^2^** |  |  |
|  |  | 0.84 | | | | | **P-value^3^** |  |  |
| 0.23 | -0.19 ± 0.81 | 1.56 ± 1.03 | | 1.75 ± 1.24 | | | **Symbiotic** | **TSH (µIU/ml)** |  |
| 0.76 | -0.11 ±1.86 | 2.16 ± 1.71 | | 2.27 ± 1.86 | | | **Placebo** |  |  |
|  | 0.83 | 0.12 | | 0.23 | | | **P-value^2^** |  |  |
|  |  | 0.72 | | | | | **P-value^3^** |  |  |
| 0.55 | -0.61 ± 5.36 | 15.49 ± 9.17 | | 16.11 ± 8.45 | | | **Symbiotic** | **Total Depression** | **Depression** |
| 0.80 | 0.29 ± 6.07 | 15.40 ± 7.83 | | 15.11 ± 9.50 | | | **Placebo** |  |  |
|  | 0.55 | 0.97 | | 0.68 | | | **P-value^2^** |  |  |
|  |  | 0.18 | | | | | **P-value^3^** |  |  |
| 0.28 | -0.93 ± 4.51 | 10.42 ± 6.25 | | 11.36 ± 6.49 | | | **Symbiotic** | **Somatic** |  |
| 0.77 | 0.23 ± 4.19 | 11.48 ± 5.52 | | 11.25 ± 6.54 | | | **Placebo** |  |  |
|  | 0.32 | 0.50 | | 0.95 | | | **P-value^2^** |  |  |
|  |  | 0.14 | | | | | **P-value^3^** |  |  |
| 0.30 | 0.40 ± 2.00 | 5.15 ± 3.64 | | 4.75 ± 3.26 | | | **Symbiotic** | **Affective** |  |
| 0.74 | 0.18 ± 2.92 | 4.04 ± 3.16 | | 3.86 ± 3.80 | | | **Placebo** |  |  |
|  | 0.74 | 0.23 | | 0.35 | | | **P-value^2^** |  |  |
|  |  | 0.61 | | | | | **P-value^3^** |  |  |
| 0.03 | 9.78 ± 22.82 | 74.25 ± 20.80 | | 64.46 ± 23.54 | | | **Symbiotic** | **Physical Function** | **Quality of life** |
| 0.22 | -4.96 ± 20.93 | 69.32 ± 24.39 | | 74.29 ± 23.08 | | | **Placebo** |  |  |
|  | 0.01 | 0.42 | | 0.12 | | | **P-value^2^** |  |  |
|  |  | 0.06 | | | | | **P-value^3^** |  |  |
| 0.21 | 11.15 ± 46.32 | 66.51 ± 37.28 | | 55.36 ± 43.76 | | | **Symbiotic** | **Role Limiting Physical Function** |  |
| 0.18 | -9.84 ± 37.95 | 62.48 ± 37.42 | | 72.32 ± 34.92 | | | **Placebo** |  |  |
|  | 0.07 | 0.69 | | 0.11 | | | **P-value^2^** |  |  |
|  |  | 0.05 | | | | | **P-value^3^** |  |  |
| 0.66 | 4.97 ± 59.45 | 53.48 ± 40.33 | | | 48.51 ± 42.67 | | **Symbiotic** | **Role Limiting Emotional Function** |  |
| 0.36 | -7.43 ± 41.87 | 59.83 ± 37.73 | | | 67.26 ± 38.62 | | **Placebo** |  |  |
|  | 0.37 | 0.54 | | | 0.09 | | **P-value^2^** |  |  |
|  |  | 0.22 | | | | | **P-value^3^** |  |  |
| 0.28 | 2.82 ± 13.64 | 58.17 ± 16.94 | | | 55.36 ± 19.00 | | **Symbiotic** | **Vitality** |  |
| 0.02 | -6.04 ± 12.69 | 53.11 ± 21.86 | | | 59.15 ± 20.37 | | **Placebo** |  |  |
|  | 0.01 | 0.36 | | | 0.47 | | **P-value^2^** |  |  |
|  |  | 0.01 | | | | | **P-value^3^** |  |  |
| 0.35 | 2.72 ± 15.29 | 60.08 ± 18.51 | | | 57.36 ± 22.16 | | **Symbiotic** | **General Mental Health** |  |
| 0.07 | -4.57 ± 12.91 | 54.89 ± 19.91 | | | 59.46 ± 19.83 | | **Placebo** |  |  |
|  | 0.06 | 0.32 | | | 0.71 | | **P-value^2^** |  |  |
|  |  | 0.02 | | | | | **P-value^3^** |  |  |
| 0.056 | 9.10 ± 24.15 | 65.80 ± 17.53 | | | 56.70 ± 23.93 | | **Symbiotic** | **Social Function** |  |
| 0.72 | -1.44 ± 20.90 | 73.29 ± 22.00 | | | 74.73 ± 22.75 | | **Placebo** |  |  |
|  | 0.09 | 0.16 | | | | 0.006 | **P-value^2^** |  |  |
|  |  | 0.03 | | | | | **P-value^3^** |  |  |
| 0.20 | 5.61 ± 22.70 | 63.03 ± 23.89 | | | 57.41 ± 24.22 | | **Symbiotic** | **Pain** |  |
| 0.10 | -6.17 ± 19.29 | 65.25 ± 25.45 | | | 71.43 ± 28.36 | | **Placebo** |  |  |
|  | 0.04 | 0.73 | | | | 0.005 | **P-value^2^** |  |  |
|  |  | 0.02 | | | | | **P-value^3^** |  |  |
| 0.02 | 7.05 ± 15.76 | 62.23 ± 15.38 | | | 55.18 ± 19.36 | | **Symbiotic** | **General Health Perception** |  |
| 0.03 | -5.93 ± 13.54 | 58.35 ± 17.20 | | | 64.29 ± 19.71 | | **Placebo** |  |  |
|  | 0.002 | 0.38 | | | 0.09 | | **P-value^2^** |  |  |
|  |  | 0.001 | | | | | **P-value^3^** |  |  |
| 0.18 | 7.97 ± 30.52 | 58.86 ± 22.81 | | | 50.89 ± 24.04 | | **Symbiotic** | **Health Change** |  |
| 0.72 | -0.99 ± 14.52 | 46.33 ± 22.26 | | | 47.32 ± 24.85 | | **Placebo** |  |  |
|  | 0.17 | 0.04 | | | 0.59 | | **P-value^2^** |  |  |
|  |  | | 0.36 | | | | **P-value^3^** |  |  |
| 0.09 | -2.24 ± 6.77 | 118.65 ± 8.82 | | | | 120.89 ± 12.77 | **Symbiotic** | **SBP (mmHg)** | **Blood pressure** |
| 0.49 | -0.55 ± 4.17 | 123.47 ± 9.01 | | | | 124.02 ± 11.31 | **Placebo** |  |  |
|  | 0.26 | 0.05 | | | | 0.34 | **P-value^2^** |  |  |
|  |  | 0.23 | | | | | **P-value^3^** |  |  |
| 0.08 | -2.06 ± 5.96 | 76.15 ± 6.13 | | | | 78.21 ± 8.95 | **Symbiotic** | **DBP (mmHg)** |  |
| 0.59 | 0.85 ± 8.23 | 81.15 ± 9.80 | | | | 80.30 ± 8.05 | **Placebo** |  |  |
|  | 0.14 | 0.03 | | | | 0.36 | **P-value^2^** |  |  |
|  |  | 0.14 | | | | | **P-value^3^** |  |  |

*1: within group comparison, paired t-test, 2: between group comparison, independent t-test, 3: two-way ANCOVA analysis with adjustment for duration of hypothyroidism, age, levothyroxine dose, and physical activity*
