# Supplementary material for: The effect of synbiotic supplementation on hypothyroidism: A randomized double-blind placebo controlled clinical trial
Source: PLoS One. 2023 Feb 6;18(2):e0277213. doi: 10.1371/journal.pone.0277213 (PMC9901790; doi:10.1371/journal.pone.0277213)
Supplement: S1 Visual abstract — (DOCX) [file pone.0277213.s006.docx]

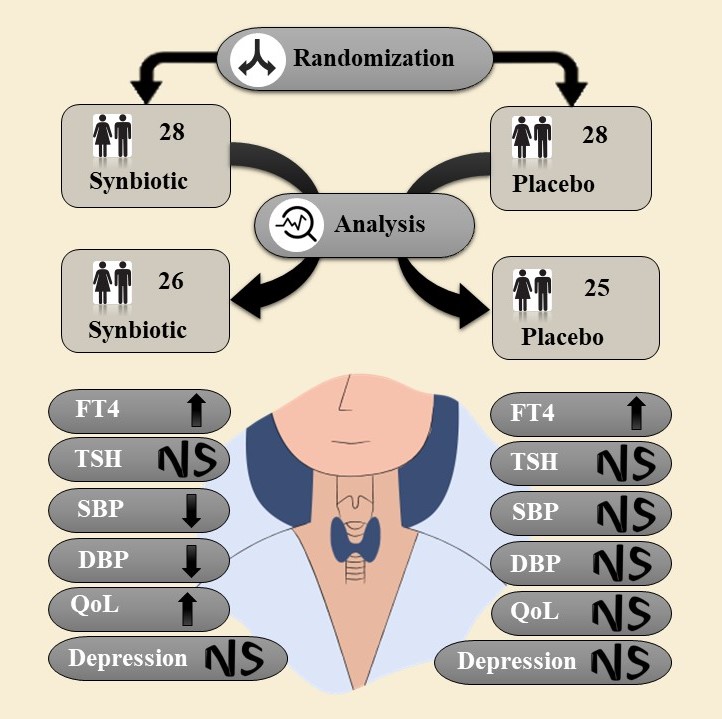
**Visual abstract:**

Overall, this study showed that synbiotic supplementation might improve serum FT4 levels, but it may be unable to change the serum level of TSH. Blood pressure and quality of life were ameliorated in the synbiotic group, while no such change was observed in the placebo group. Depression was changed in neither of the groups following this intervention.

NS: non-significant.
